# Spatially restricted substrate-binding site of cortisol-synthesizing CYP11B1 limits multiple hydroxylations and hinders aldosterone synthesis

**DOI:** 10.1016/j.crstbi.2021.08.001

**Published:** 2021-08-26

**Authors:** Kuniaki Mukai, Hiroshi Sugimoto, Katsumasa Kamiya, Reiko Suzuki, Tomomi Matsuura, Takako Hishiki, Hideo Shimada, Yoshitsugu Shiro, Makoto Suematsu, Norio Kagawa

**Affiliations:** a Department of Biochemistry, Keio University School of Medicine, Shinjuku, Tokyo 160-8582, Japan; b Medical Education Center, Keio University School of Medicine, Shinjuku, Tokyo 160-8582, Japan; c Clinical and Translational Research Center, Keio University School of Medicine, Shinjuku, Tokyo 160-8582, Japan; d Synchrotron Radiation Life Science Instrumentation Team, RIKEN SPring-8 Center, Sayo, Hyogo 679-5148, Japan; e Center for Basic Education and Integrated Learning, Kanagawa Institute of Technology, Atsugi, Kanagawa 243-0292, Japan; f Graduate School of Life Science, University of Hyogo, Kamigori, Hyogo 678-1297, Japan; g Office of CIBoG, Nagoya University Graduate School of Medicine, Nagoya, Aichi 466-8550, Japan

**Keywords:** Adrenal, Cytochrome P450, Molecular dynamics, Steroid hormone, X-ray crystallography, DOC, 11-deoxycorticosterone, DOF, 11-deoxycortisol

## Abstract

Human cytochromes P450_11β_ (CYP11B1) and P450_aldo_ (CYP11B2) are monooxygenases that synthesize cortisol through steroid 11β-hydroxylation and aldosterone through a three-step process comprising 11β-hydroxylation and two 18-hydroxylations, respectively. CYP11B1 also catalyzes 18-monohydroxylation and 11β,18-dihydroxylation. To study the molecular basis of such catalytic divergence of the two enzymes, we examined a CYP11B1 mutant (Mt-CYP11B1) with amino acid replacements on the distal surface by determining the catalytic activities and crystal structure in the metyrapone-bound form at 1.4-Å resolution. Mt-CY11B1 retained both 11β-hydroxylase and 18-hydroxylase activities of the wild type (Wt-CYP11B1) but lacked 11β,18-dihydroxylase activity. Comparisons of the crystal structure of Mt-CYP11B1 to those of Wt-CYP11B1 and CYP11B2 that were already reported show that the mutation reduced the innermost space putatively surrounding the C3 side of substrate 11-deoxycorticosterone (DOC) bound to Wt-CYP11B1, while the corresponding space in CYP11B2 is enlarged markedly and accessible to bulk water through a channel. Molecular dynamics simulations of their DOC-bound forms supported the above findings and revealed that the enlarged space of CYP11B2 had a hydrogen bonding network involving water molecules that position DOC. Thus, upon positioning 11β-hydroxysteroid for 18-hydroxylation in their substrate-binding sites, steric hindrance could occur more strongly in Mt-CYP11B1 than in Wt-CYP11B1 but less in CYP11B2. Our investigation employing Mt-CYP11B1 sheds light on the divergence in structure and function between CYP11B1 and CYP11B2 and suggests that CYP11B1 with spatially-restricted substrate-binding site serves as 11β-hydroxylase, while CYP11B2 with spatially-extended substrate-binding site successively processes additional 18-hydroxylations to produce aldosterone.

## Highlights

• CYP11B1 and CYP11B2 catalyze steroid hydroxylation for syntheses of cortisol and aldosterone, respectively.
• Structural basis for their differences in ability of multiple hydroxylations remains unclear.
• A CYP11B1 mutant generated is characterized by monohydroxylase activities.
• X-ray crystallography and molecular dynamics simulation reveal spatial restriction in substrate-binding site of the mutant.
• Spatial dimension of the substrate-binding sites is crucial for differential production of gluco- and mineralocorticoids.

## Graphical abstract

**Figure:**
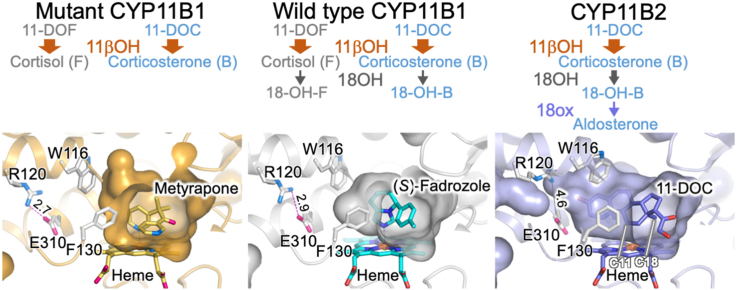

## 1 Introduction

Glucocorticoids regulate homeostasis of energy metabolism and stress and immune responses, and mineralocorticoids regulate homeostasis of water and electrolytes. The two classes of adrenal corticosteroids are synthesized from cholesterol by a series of enzymes including cytochromes P450 that catalyze various reactions through monooxygenation (Miller and Auchus, 2011; Stewart and Newell-Price, 2015). The last step of the synthetic pathway of the major glucocorticoids, cortisol in human and corticosterone in rodents, is catalyzed by steroid 11β-hydroxylase cytochrome P450_11β_ (CYP11B1), while those of the potent mineralocorticoid, aldosterone, are catalyzed by aldosterone synthase cytochrome P450_aldo_ (CYP11B2) (Fig. S1). They are a pair of closely-related cytochromes P450, and their amino acid sequences of the mature enzymes share 94% identities to each other (Fig. S2). As their physiological functions, CYP11B1 converts 11-deoxycortisol (DOF) and 11-deoxycorticosterone (DOC) to cortisol and corticosterone, respectively, while CYP11B2 catalyzes, in addition to 11β-hydroxylation of DOC, 18-hydroxylation and 18-oxidation to yield aldosterone (Fig. S1). The two enzymes are expressed in the different adrenocortical cell-zones and in adrenocortical adenomas in humans (Nishimoto et al., 2010). The physiological production of the gluco- and mineralocorticoid in humans and some other mammals including rodents depends on the differences in the catalytic activities of the two enzymes and in their cell-specific expression (Miller and Auchus, 2011; Nishimoto et al., 2010; Stewart and Newell-Price, 2015).

Previous studies have explored similarities and differences in the catalytic functions of CYP11B1 and CYP11B2 using transfected cells expressing them (Curnow et al., 1991, 1997; Kawamoto et al., 1992; Mulatero et al., 1998) or purified human CYP11B enzymes (Hobler et al., 2012; Strushkevich et al., 2013; Zöllner et al., 2008). As described in Table S1 (see columns “Wild type”), both enzymes function primarily as 11β-hydroxylase and secondarily as 18-hydroxylase. Additionally, CYP11B1 has a low activity of 11β,18-dihydroxylase, whereas CYP11B2 has a higher activity of the dihydroxylase and further catalyzes 18-oxidation (Curnow et al., 1991, 1997; Hobler et al., 2012; Kawamoto et al., 1992; Mulatero et al., 1998; Strushkevich et al., 2013). Thus, the key feature differentiating between the two enzymes is the capability to process the successive 18-hydroxylation following 11β-hydroxylation.

Kinetic studies on the processivity of aldosterone formation were carried out using purified bovine enzyme. The results revealed that DOC is converted successively by 11β-hydroxylation and then twice by 18-hydroxylation into aldosterone, though most of the intermediate products dissociate (Ikushiro et al., 1989; Imai et al., 1998; Kominami et al., 1994). These catalytic properties of bovine aldosterone-synthesizing enzyme well explain the known catalytic activities of human CYP11B2.

Recently, a crystal structure of human CYP11B1 complexed with (*S*)-enantiomer of fadrozole, the racemic form of which is known as an inhibitor of estrogen-producing aromatase CYP19A1, was reported (Brixius-Anderko and Scott, 2019). The overall structure is very similar to the crystal structures of CYP11B2 complexed with DOC or (*R*)-fadrozole (Strushkevich et al., 2013). Amino acid residues forming the substrate-binding site of the two enzymes are identical to each other, and each of the residues is located at slightly different positions between the two enzymes (Brixius-Anderko and Scott, 2019; Strushkevich et al., 2013). The crystal structure of DOC-bound CYP11B2 shows that the C11 atom of DOC is located within the distance of monooxygenation by heme-bound active oxygen. For the subsequent reaction at C18, 3.3 ​Å distant from C11, the processivity depends on the capability to bind 11β-hydroxy steroid whose C18 is close enough to the iron-bound oxygen species. Although the selective binding of the different enantiomers to the two isoforms indicates the divergence in their substrate-binding sites, the structural elements for the isoform-specific configurations of the sites which dictate the differential catalytic functions have remained unclear.

To provide the structural basis for the differentiation between CYP11B1 and CYP11B2, we focus on a novel CYP11B1 mutant that retains both 11β- and 18-monohydroxylase activities comparable to those of the wild type but is deficient in 11β,18-dihydroxylase activity. The mutant is generated in our attempt to increase in protein hydrophilicity for crystallography by replacing hydrophobic residues with hydrophilic ones at six positions on the distal surface putatively associating with mitochondrial membranes (Fig. S2, residues in red). Since this mutation decouples the two successive monohydroxylations, we have solved the X-ray crystal structure of the mutant complexed with metyrapone, a classical inhibitor of 11β-hydroxylase, at 1.4-Å resolution. Subsequently, we performed molecular dynamics (MD) studies using the X-ray data of the CYP11B1 mutant as well as of the wild types of CYP11B1 and CYP11B2 to generate substrate-bound forms of them and examine the structural differences in the substrate-binding site among them. These comparative analyses among the three enzymes reveal that the CYP11B1 mutant has structural alterations in the substrate-binding site along with the deficiency in 11β,18-dihydroxylase activity. The novel mutant has a key role in highlighting the differences in structure and function between CYP11B1 and CYP11B2. The present observations suggest structural bases for the catalytic divergence in the regioselective and successive reactions of the CYP11B enzymes that differentially produce glucocorticoids and mineralocorticoids.

## 2 Results

### 2.1 Spectroscopic and ligand-binding analyses of wild type and mutant of CYP11B1

The purified CYP11B1 proteins (Fig. S3), both the wild type and the mutant, exhibited typical UV–visible absorption spectra of ferric cytochromes P450 with the Soret peak at 417.5 ​nm in the absence of substrates (Figs. S4a and b, *solid lines*). Addition of the substrate DOC at 400 ​μM to both shifted the Soret peak to 392 ​nm (Figs. S4a and b, *dashed lines*), and addition of DOF also induced similar spectral changes. These spectral data indicate that binding of the substrates displaces heme-bound water, changing six coordinated low-spin state to five coordinated high-spin state. Sodium dithionite partially reduced the substrate-bound enzymes (Figs. S4c and d, *dotted lines*), and formation of ferrous CO complexes (peak at 447.5 ​nm) was observable (Figs. S4c and d, *dashed-dotted lines*). When a detergent Cymal-5 was added at 5 ​mM to the substrate-free ferric enzymes, both were found to be converted into high-spin state of the heme iron (Figs. S4e and f, *dashed lines*; see Materials and Methods). In the presence of Cymal-5 the wild type was converted to fully reduced CO form (Fig. S4e, *dashed-dotted line*), while the mutant was converted to ferrous CO form at a higher level than in the presence of DOC although formation of P420 was detected as a bump at 420 ​nm (Fig. S4f, *dashed-dotted line*). Partial reduction of heme iron by sodium dithionite may be characteristic of the CYP11B enzymes as described previously (Hobler et al., 2012; Zöllner et al., 2008).

Affinities of the two enzymes for the substrates DOC or DOF and other ligands were determined by titration assays using the ferric forms. Addition of increasing concentrations of DOF and DOC caused characteristic difference spectral changes in saturable manners (Figs. S5a–d). Dissociation constant (*K*_d_) values of the wild type for DOF and DOC were 21.2 ​μM and 15.1 ​μM, respectively, and those of the mutant for DOF and DOC were 3.3 ​μM and 3.8 ​μM, respectively (Table 1). Addition of cortisol or corticosterone, the products of 11β-hydroxylation, at up to 1.6 ​mM did not induce spectral changes of both substrate-free enzymes, and their presence at 1 ​mM did not repress binding of DOC or DOF, indicating that the primary products of the enzymatic reaction did not bind to both ferric enzymes under the present conditions.

**Table 1:** Binding of steroid or ligand and steady-state kinetics.

				Wild type		Mutant	
Binding							
	Steroid or ligand		Spectral shift	*K*_d_ (μM)		*K*_d_ (μM)	

	11-Deoxycortisol (DOF)		Type I	21.2 ​± ​1.4		3.31 ​± ​0.13	
	11-Deoxycorticosterone (DOC)		Type I	15.1 ​± ​1.2		3.81 ​± ​0.16	
	Metyrapone		Type II	0.0361 ​± ​0.0011^a^		0.0119 ​± ​0.0005^a^	

Steady-state kinetics							
	Substrate	Activity	Product	*k*_cat_ (/min)	*K*_m_ (μM)	*k*_cat_ (/min)	*K*_m_ (μM)
	DOF	11β-OHase	Cortisol (F)	129.4 ​± ​7.2	28.2 ​± ​3.6	90.56 ​± ​2.79	10.5 ​± ​1.0
		18-OHase	18OH-DOF	nt^b^	nt^b^	nt^b^	nt^b^
		11β,18-diOHase	18OH–F	0.214 ​± ​0.023	38.7 ​± ​8.6	0	na^c^
		11β,18-diOHase,18-oxidase	18oxo-F	nt^b^	nt^b^	nt^b^	nt^b^

	DOC	11β-OHase	Corticosterone (B)	161.0 ​± ​4.5	17.7 ​± ​1.3	132.7 ​± ​3.9	7.77 ​± ​0.77
		18-OHase	18OH-DOC	7.94 ​± ​0.24	20.2 ​± ​1.6	5.76 ​± ​0.15	8.42 ​± ​0.73
		11β,18-diOHase	18OH–B	0.650 ​± ​0.055	40.5 ​± ​7.0	0	na^c^
		11β,18-diOHase,18-oxidase	Aldosterone	0	na^c^	0	na^c^

DOF, 11-Deoxycortisol; F, Cortisol; DOC, 11-Deoxycorticosterone; B, Corticosterone.

Values are shown as mean ​± ​standard error (n ​= ​3, binding assays; n ​= ​6, enzyme reaction assays).

a Calculated from apparent *K*_d_ values obtained by titration with metyrapone in the presence of 320 ​μM DOC.

b nt, not tested.

c na, not assigned.

The *K*_d_ values of the wild type and the mutant enzymes for metyrapone, a classical inhibitor of 11β-hydroxylase, were evaluated to be 36.1 ​nM and 11.9 ​nM, respectively (Table 1), from apparent values obtained in the presence of a saturating concentration of 320 ​μM DOC as competitor. The difference spectra obtained in the titrations correspond to the spin-type shift from DOC-bound high-spin to metyrapone-bound low-spin (Figs. S5e and f). Addition of metyrapone to the substrate-free ferric enzymes shifted the Soret peak from 417.5 ​nm to 421.5 ​nm. These observations were consistent with the nitrogen coordination to the heme iron known as type-II spectral shift. Thus, the affinities of the mutant for the substrates and the nitrogen coordinating ligands are higher than those of the wild type.

### 2.2 The CYP11B1 mutant catalyzes 11β- or 18-monohydroxylation but not 11β,18-dihydroxylation

**Fig. 1:**
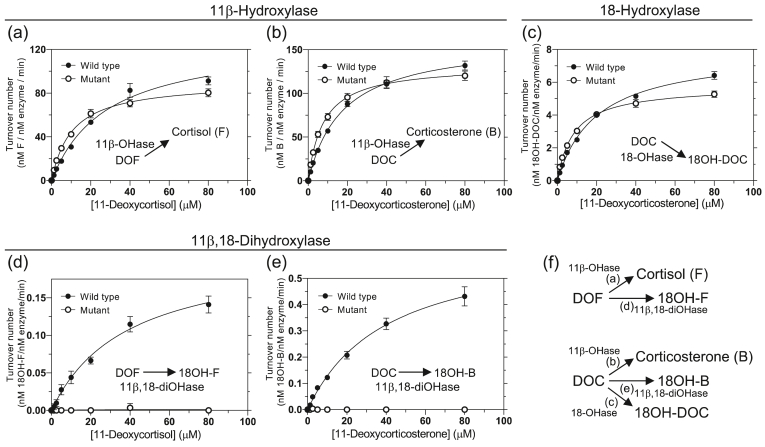
Steady-state kinetics of 11β-hydroxylase, 18-hydroxylase, and 11β,18-dihydroxylase activities of the CYP11B1 wild type and the mutant. Conversion of substrate 11-deoxycortisol (DOF) into cortisol (F) and 18-hydroxy cortisol (18OH–F) and conversion of 11-deoxycorticosterone (DOC) into corticosterone (B), 18-hydroxy DOC (18OH-DOC), and 18-hydroxyl corticosterone (18OH–B) were assayed. (a and b) 11β-Hydroxylation convers DOF and DOC into cortisol (F) and corticosterone (B), respectively. (c) 18-Hydroxylation convers DOC into 18-hydroxy DOC (18OH-DOC). (d and e) 11β,18-Dihydroxylation convers DOF and DOC into 18-hydroxyl cortisol (18OH–F) and 18-hydroxyl corticosterone (18OH–B), respectively. Reaction mixture contained 0–80 ​μM DOF or DOC, 2 ​nM CYP11B1, 0.125 ​μM adrenodoxin reductase, 5 ​μM adrenodoxin, and NADPH regenerating system. Products were analyzed using liquid chromatography-tandem mass spectrometry. Panel (f) depicts the activities assayed in panels (a)–(e). Values of mean ​± ​standard error obtained from 6 separate experiments are shown. Closed and open circles indicate values obtained with the wild type and the mutant, respectively.

To compare the catalytic functions between the mutant and the wild type, the three activities, 11β-hydroxylase, 18-hydroxylase, and 11β,18-dihydroxylase, of both enzymes were assayed using DOF and DOC as substrates with a reconstituted electron transfer chain. The products converted from the substrates were analyzed except for 18-hydroxy DOF since its standard chemical was unavailable (Table S2). Plots of the turnover numbers vs the substrate concentrations are shown in Fig. 1, and the kinetic parameters (*k*_cat_ and *K*_m_) are summarized in Table 1.

First, the wild type catalyzed primarily 11β-hydroxylation of DOF to cortisol (*k*_cat_ 129.4 min^−1^, *K*_m_ 28.2 ​μM; Fig. 1a, *closed circle*) and of DOC to corticosterone (*k*_cat_ 161.0 min^−1^, *K*_m_ 17.7 ​μM; Fig. 1b, *closed circle*). Similarly, the mutant catalyzed 11β-hydroxylation of DOF to cortisol (*k*_cat_ 90.6 min^−1^, *K*_m_ 10.5 ​μM; Fig. 1a, *open circle*) and of DOC to corticosterone (*k*_cat_ 132.7 min^−1^, *K*_m_ 7.8 ​μM; Fig. 1b, *open circle*).

Second, the wild type catalyzed secondarily 18-hydroxylation of DOC to 18-hydroxy DOC (*k*_cat_ 7.9 min^−1^, *K*_m_ 20.2 ​μM; Fig. 1c, *closed circle*). The mutant also catalyzed 18-hydroxylation of DOC to 18-hydroxy DOC (*k*_cat_ 5.8 min^−1^, *K*_m_ 8.4 ​μM; Fig. 1c, *open circle*). Thus, the *k*_cat_ values of the mutant for both 11β- and 18-monohydroxylase activities were slightly lower by 0.7–0.8-fold than those of the wild type. On the other hand, the *K*_m_ values of the mutant for DOF in 11β-hydroxylation, DOC in 11β-hydroxylation, and DOC in 18-hydroxylation decreased by 2.3–2.7-fold compared to those of the wild type, being parallel with the decreases in the *K*_d_ values of the mutant for both substrates in the binding assays (Figs. S5a–d and Table 1).

Third, the wild type catalyzed very slowly 11β,18-dihydroxylation of DOF to 18-hydroxy cortisol (*k*_cat_ 0.21 min^−1^, *K*_m_ 38.7 ​μM; Fig. 1d, *closed circle*) and of DOC to 18-hydroxy corticosterone (*k*_cat_ 0.65 min^−1^, *K*_m_ 40.5 ​μM; Fig. 1e, *closed circle*). By contrast, the mutant did not produce 18-hydroxy cortisol and 18-hydroxy corticosterone from DOF and DOC, respectively (Fig. 1d and e , *open circle*, and Fig. S6). Lastly, aldosterone was not produced from DOC by both enzymes. Therefore, the replacement of the amino acid residues caused a complete lack of the activity of 11β,18-dihydroxylase without marked effect on the two monohydroxylase activities.

For comparison of catalytic efficiencies (*k*_cat_/*K*_m_) of the monooxygenations at 11β and 18 between the mutant and the wild type, the data obtained above were used for calculation. The mutant was found to have almost two-fold higher catalytic efficiencies of the monooxygenations at both 11β and 18 compared to those of the wild type. This evaluation suggests that the mutation did not alter the structures essential for catalysis of monooxygenation.

### 2.3 Crystal structure of the CYP11B1 mutant reveals spatial restriction in the substrate-binding site

#### 2.3.1 Overall structure

**Fig. 2:**
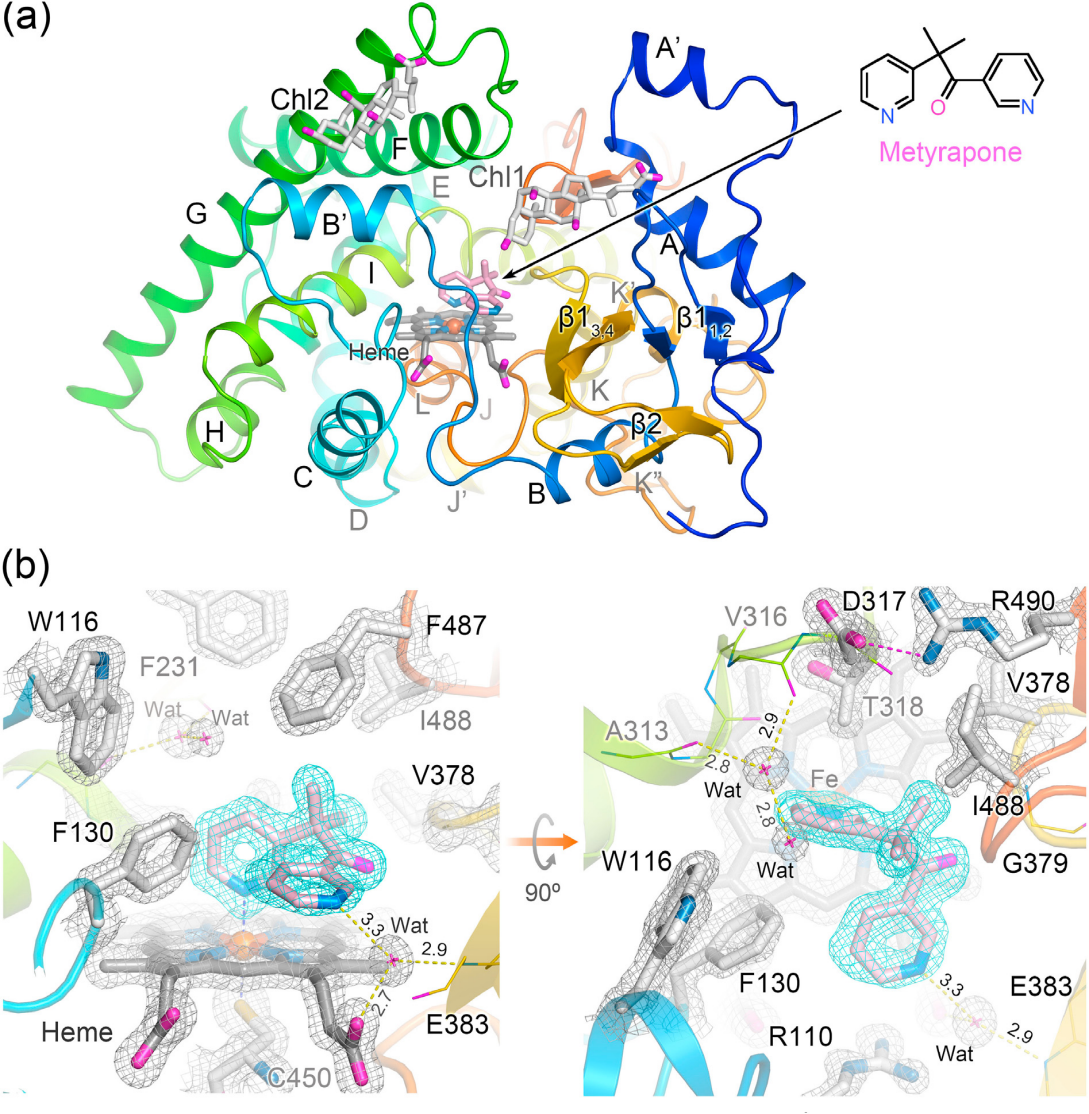
Overall crystal structure of CYP11B1 mutant determined at 1.4 ​Å resolution and binding of metyrapone. (a) The overall structure is shown by ribbon representation in rainbow colors from N-terminus (blue) to C-terminus (orange). The heme (gray), metyrapone (pink), and two molecules of cholate (Chl1 and Chl2) are shown in stick model. Helices are labeled alphabetically, and sheets by β with numbers (see Fig. S7). (b) The omit map for metyrapone (blue mesh) in the substrate-binding site is contoured at 4.5 σ level. The 2*F*_o_-*F*_c_ map (gray mesh) of side chains of amino acid residues, heme, and waters are contoured at 2.0 σ level. Hydrogen bond interactions are indicated by yellow dotted lines with a distance (Å) between hydrogen bond donor and acceptor, and an ionic bond between the side chains of D317 and R490 is indicated by magenta dotted line.

The crystal structure of the CYP11B1 mutant complexed with metyrapone was determined at a resolution of 1.4 ​Å (Fig. 2 and Table 2). The asymmetric unit of the crystals has a single protein molecule, and two molecules of cholate are co-crystallized exposing their β-face to solvent (Fig. 2a). The nitrogen atom in 2-pyridinyl of metyrapone coordinates to the heme iron (Fig. 2b), being consistent with the low-spin state (Fig. S5f). The other nitrogen atom in 1-pyridinyl seems to interact with a water molecule which forms hydrogen bonds to a propionate of the heme and the main chain amide nitrogen of E383 (Fig. 2b). Two molecules of water are located near the roof of the substrate-binding site and form a hydrogen bonding network with the carbonyl oxygens of A313 and V316 (Fig. 2b). The overall structure of the CYP11B1 mutant retains a canonical cytochrome P450 fold (Fig. 2a and Fig. S7).

**Table 2:** X-ray data collection and refinement statistics.

	CYP11B1 mutant (Metyrapone complex)
**Data collection**	
Beamline (SPring-8)	BL41XU
Space group	*P*2_1_2_1_2_1_
Wavelength (Å)	1.0
Cell dimensions, *a, b, c* (Å)	75.18, 84.60, 85.61
Resolution (Å) a	50–1.40 (1.48–1.40)
Observed reflections	1816384
Unique reflections	105301
Average *I/*σ *(I)*^a^	29.3 (2.1)
Completeness (%) a	97.6 (90.0)
Redundancy a	17.2 (14.4)
CC_1/2_	100.0 (76.3)
*R*_meas_ (%) a^*,*^^b^	4.6 (117.4)
Wilson *B*-factor (Å^2^)	30.7
**Refinement**	
*R*_work_ (%) c	14.0
*R*_free_ (%) c	16.3
Average *B* value (Å^2^)	28.9
R.m.s.d bond (Å)	0.008
R.m.s.d angles (°)	1.53
Ramachandran plot	
Favored region (%)	97.0
Outlier regions (%)	0.0
PDB entry	7E7F

a Values in parentheses are for the highest-resolution shell.

b *R*_meas_ ​= ​Σ_*hkl*_{*n*/(*n*-1)}^1/2^ Σ_*i*_|*I*_*i*_(*hkl*)-<*I*(*hkl*)>|/Σ_*hkl*_Σ_*i*_*I*_*i*_(*hkl*), where *n* is the multiplicity of reflection *hkl*, and <*I*(*hkl*)> is the average intensity of *i* observations.

c *R*_work_ ​= ​Σ_*hkl*_|*F*_obs_(*hkl*) - *F*_calc_(*hkl*)|/Σ_*hkl*_*F*_obs_(*hkl*), where *F*_obs_ and *F*_calc_ are the observed and calculated structure factors, respectively. *R*_free_ was calculated with 5% of the reflections.

**Fig. 3:**
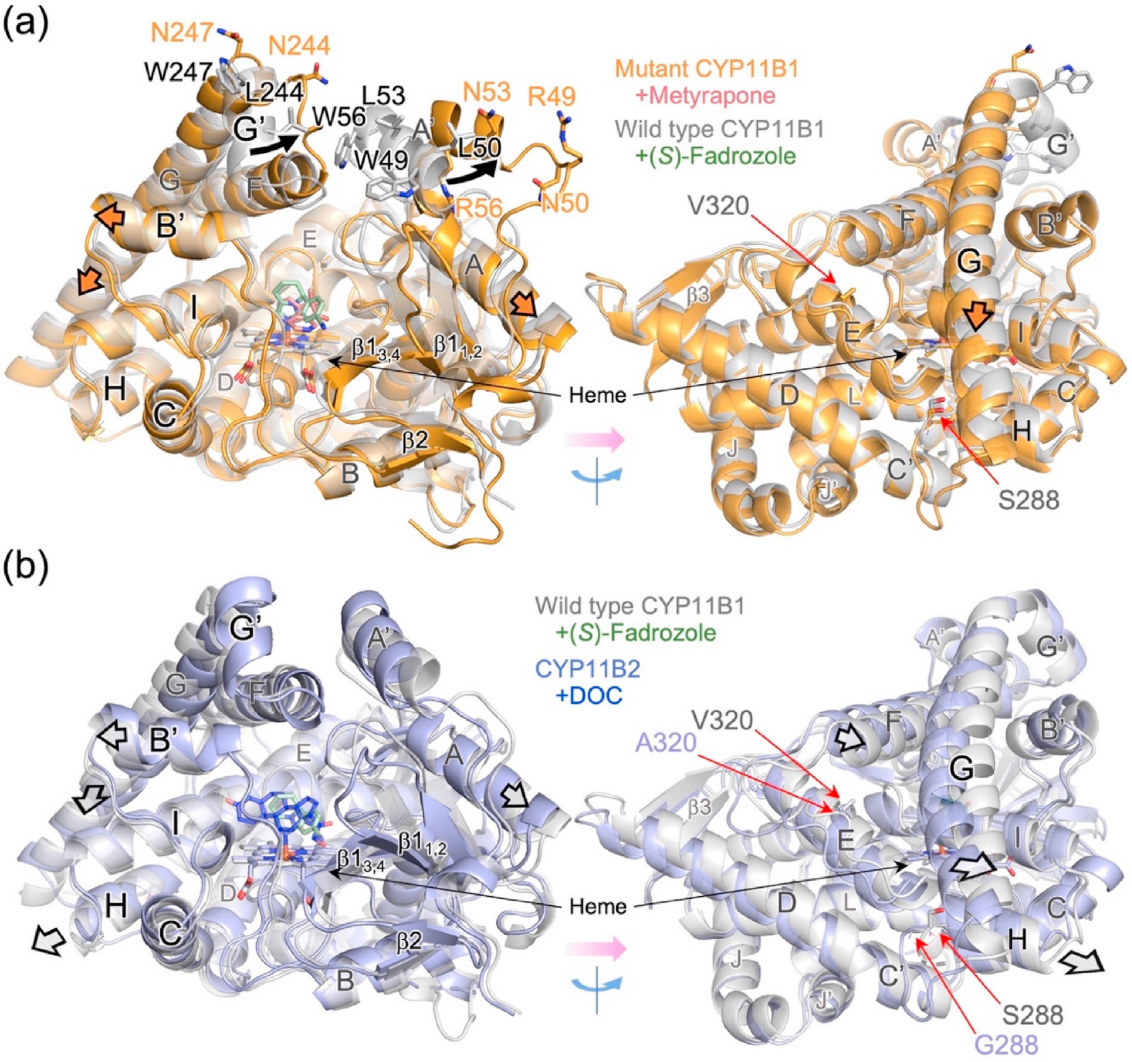
Comparison of the overall structures among three CYP11B enzymes. (a) Superposition of metyrapone-bound CYP11B1 mutant (orange) and (*S*)-fadrozole-bound CYP11B1 wild type (gray; PBD ID 6M7X). The two crystal structures are aligned pairwise at 38 atoms (heme iron, porphyrin skeleton, and 13 α-carbon atoms of residues 441–453) and shown by ribbon representation. Side chains of the amino acid residues subjected to the replacement at positions 49, 50, 53, 56, 244, and 247 on the distal surface of CYP11B1 are indicated by orange (mutant) and gray (wild type) sticks. Structural changes that occur in helices A′ and G′ with the amino acid replacements are indicated by black arrows. Metyrapone and (*S*)-fadrozole are indicated by pink and light green sticks, respectively. Positional shifts of the main chain, such as helices A, B′, and G, in the CYP11B1 mutant are indicated by thick orange arrows. (b) Superposition of (*S*)-fadrozole-bound CYP11B1 wild type (gray) and DOC-bound CYP11B2 (light blue; PDB ID 4DVQ). The two crystal structures are aligned using the same procedures as those for (a). (*S*)-Fadrozole and DOC are indicated by light green and blue sticks, respectively. Positional shifts of the main chain, such as helices A, B′, F, G, and H, in the CYP11B1 wild type compared to the structure of CYP11B2 are indicated by thick gray arrows. Residues S288 and V320 of CYP11B1 and residues G288 and A320 of CYP11B2 are indicated by red arrows in the right panels of (a) and (b), respectively. They are the isoform-specific residues that have been reported to be critical for the functional differentiation of CYP11B1 and CYP11B2 (Curnow et al., 1997) (see also Fig. S11). Helices and sheets are labeled as in Fig. 2a.

**Fig. 4:**
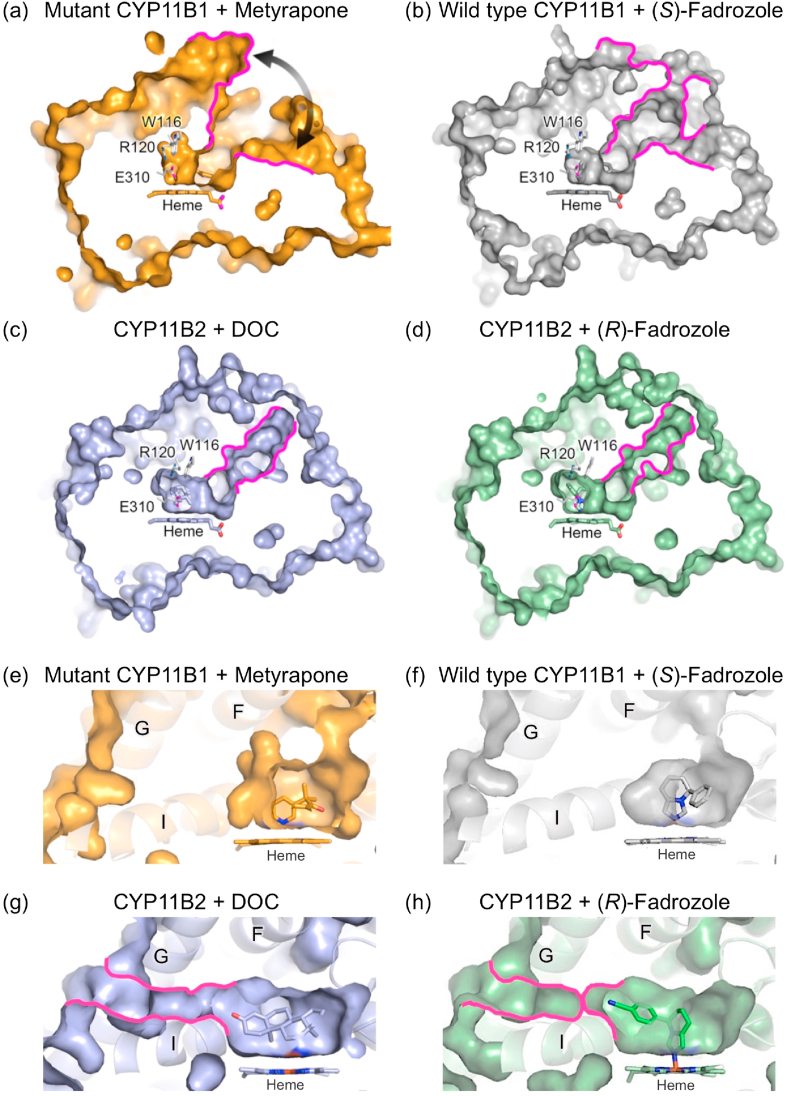
Molecular surface analysis of channels. (a–d) Surface representations with a cross-section passing through the substrate access channel indicated by tracing in magenta. (a) CYP11B1 mutant bound to metyrapone with an open cleft indicated by a curved arrow. (b) CYP11B1 wild type bound to (*S*)-fadrozole (gray; PBD ID 6M7X). (c) CYP11B2 bound to DOC (light blue; PDB ID 4DVQ). (d) CYP11B2 bound to (*R*)-fadrozole (light green; PDB ID 4FDH). The substrate-binding sites in the four structures are accessible to bulk water through their substrate access channels. Ligands, steroid, heme, and side chains of W116, R120, and E310 are indicated in stick. (e–h) Surface representations with a cross-section passing through the substrate-binding site. Absence of a putative water channel in (e) the CYP11B1 mutant bound to metyrapone and (f) the CYP11B1 wild type bound to (*S*)-fadrozole. Presence of a putative water channel in (g) CYP11B2 bound to DOC as indicated by tracing in magenta, while the channel is closed in (h) CYP11B2 bound to (*R*)-fadrozole. Molecular surfaces are calculated with a probe radius of 1.4 ​Å using PyMOL.

To compare the structure of the mutant with that of the wild type complexed with (*S*)-fadrozole (Brixius-Anderko and Scott, 2019), the heme iron, the porphyrin skeleton, and the α-carbons of the proximal loop including the fifth ligand cysteine residue were employed to superpose. The alignment shows two major structural shifts on the distal surface of the mutant (Fig. 3a, *black arrows*). First, the amino acid replacements at the four residues (W49R, L50N, L53N, and W56R) in helix A′ of the wild type caused a shift of the helix together with a shift of the turn from β1-1 to β1-2. Second, the replacements at the two residues (L244N and W247N) in helix G′ of the wild type caused uncoiling in part and a shift of it. Along with the structural shifts, the substrate access channel has an open entrance (Fig. 4a). One of two cholate molecules is located on the cleft, and the other is on the position corresponding to helix G’ of the wild type (Fig. 2a). The crystal structures of both wild type enzymes, CYP11B1 and CYP11B2, do not have such an open cleft, while their substrate-binding sites are accessible to bulk water through the substrate access channel that allows passage of water molecules (Fig. 4b–d) (Brixius-Anderko and Scott, 2019; Strushkevich et al., 2013). The noticeable structural changes that occurred in the mutant are limited to the distal surface and the substrate access channel.

Detailed comparison of the crystal structures between the CYP11B1 mutant and the wild-type showed that the mutation caused helices A, B’, F, G, and I and some loops to shift evidently outward from or downward to the heme (Fig. 3a left, *thick arrows* and Supplementary movie S1). A lateral view shows that helix G notably shifts downward (Fig. 3a right*, thick arrow*). Similar structural shifts as mentioned above were detected when the crystal structure of the (*S*)-fadrozole-bound wild type CYP11B1 was superposed on that of DOC-bound CYP11B2 (Fig. 3b left, *thick arrows*). Helices F, G, and H of the wild type of CYP11B1 in comparison to those of CYP11B2 exhibit characteristic shifts (Fig. 3b right, thick arrows). The structural shifts could involve the isoform-specific residues at 288 and 320 (Fig. 3b, right), as discussed later (see also Fig. S11), which were previously shown to be crucial for determining the different abilities of CYP11B1 and CYP11B2 in the successive 18-hydroxylations (Table S1) (Curnow et al., 1997). These comparisons indicate that the structural differences between CYP11B1 and CYP11B2 are enlarged by the mutation. The observed shifts are consistent with more marked reduction in the space putatively surrounding the C3 side of steroid bound to the CYP11B1 mutant compared to the wild type, as described below.

#### 2.3.2 Substrate-binding site

**Fig. 5:**
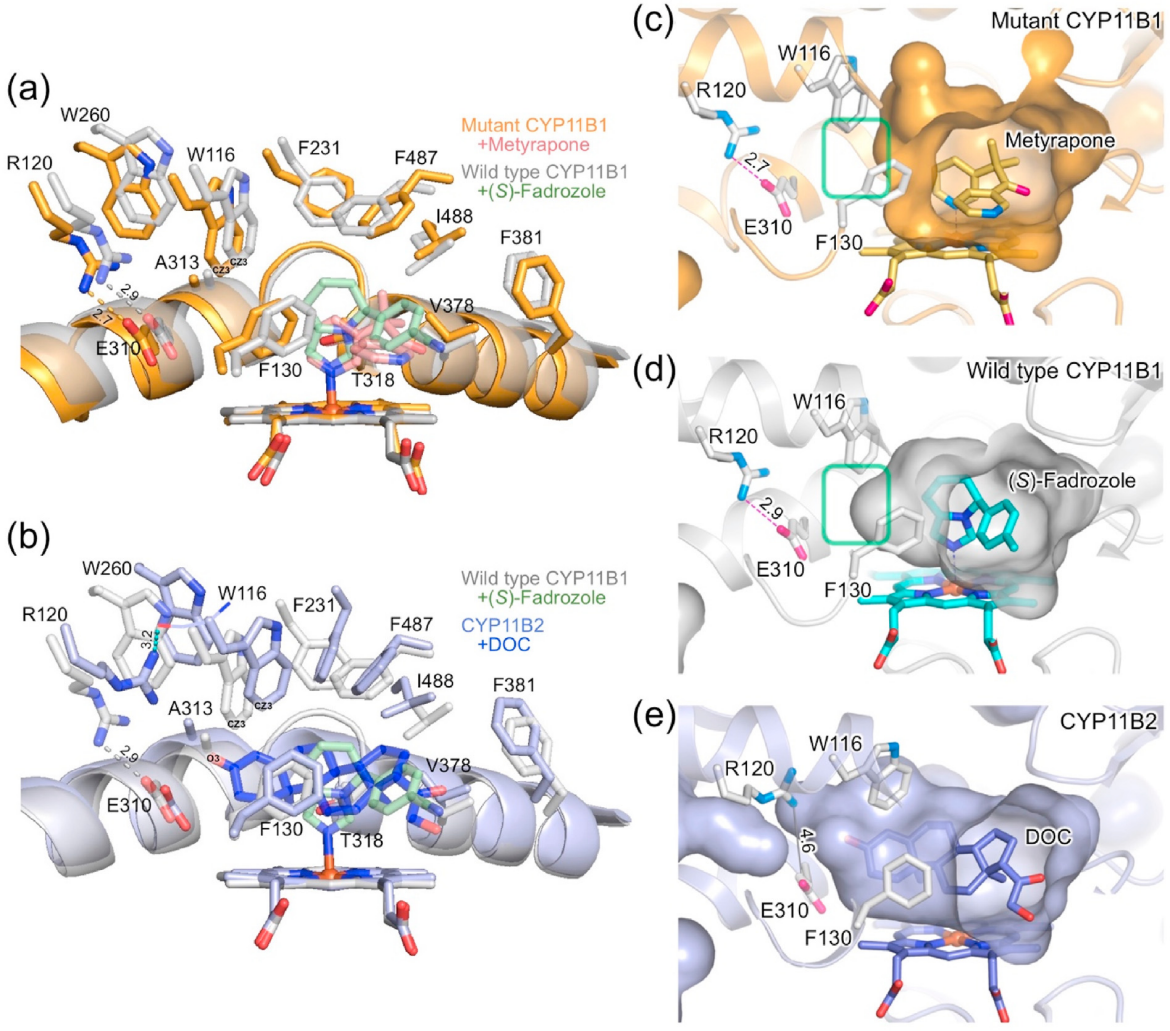
Comparison of the substrate-binding site among the three enzymes. (a and b) Location of amino acid residues forming the substrate-binding sites. (a) Superposition of metyrapone-bound CYP11B1 mutant (orange) and (*S*)-fadrozole-bound CYP11B1 wild type (gray; PBD ID 6M7X). (b) Superposition of (*S*)-fadrozole-bound CYP11B1 wild type (gray) and DOC-bound CYP11B2 (light blue; PDB ID 4DVQ). The two crystal structures in each panel are aligned in the same manner as those in Fig. 3. Side chains of the residues W116, R120, F130, F231, W260, E310, A313, T318, V378, F381, F,487, and I488 are shown in sticks. Helix I behind the substrate-binding site is shown by ribbon representation. Ionic bonds between the side chains of R120 and E310 in both CYP11B1s and a hydrogen bond interaction between the side chain of R120 and the main chain carbonyl oxygen of W116 in CYP11B2 are indicated by dashed lines with distance (Å). Cζ3 atoms of the side chain of W116 are labeled as CZ3 in panels (a) and (b), and C3 carbonyl oxygen atom of DOC is labeled as O3 in panel (b). Metyrapone, (*S*)-fadrozole, and DOC are shown in pink, light green, and blue, respectively. (c–e) Molecular surface representations of the substrate-binding site. (c) Metyrapone-bound CYP11B1 mutant, (d) (*S*)-fadrozole-bound CYP11B1 wild type, and (e) DOC-bound CYP11B2. The innermost surface (left-hand area indicated by green box) in the substrate-binding site of (c) metyrapone-bound CYP11B1 mutant does not expand in comparison to that of (d) (*S*)-fadrozole-bound wild type. The innermost surface in the site of (e) DOC-bound CYP11B2 expands toward the space near the side chains of R120 and E310. Ionic bond is not formed between the side chains of R120 and E310 as indicated by a solid line with a distance of 4.6 ​Å in panel (e). Molecular surfaces are calculated with a probe radius of 1.6 ​Å using PyMOL.

The substrate-binding site of the metyrapone-bound CYP11B1 mutant is formed by the residues W116, R120, F130, F231, W260, E310, A313, G314, T318, V378, F381, F487, and I488 (Fig. 5a, *orange*). The same set of residues forms the substrate-binding site of the (*S*)-fadrozole-bound CYP11B1 wild type (Brixius-Anderko and Scott, 2019) (Fig. 5a and b, *gray*) and DOC-bound CYP11B2 (Strushkevich et al., 2013) (Fig. 5b, *light blue*). To examine the spatial configuration of their substrate-binding sites, molecular surfaces were calculated with a probe radius of 1.6 ​Å. The surface representations delineate differences in geometry among them (Fig. 5c–e). The innermost space, which is formed by the residues including W116, R120, F130, and E310, is reduced in the mutant compared to the CYP11B1 wild type (Fig. 5c *vs* d, *green boxes*), whereas CYP11B2 has an enlarged space which surrounds the C3 carbonyl side of DOC (Fig. 5e).

These spatial differences are attributable mainly to two features. First, the side chains of R120 and E310 form an ionic bond in both CYP11B1 (Fig. 5a, c and d) but not in CYP11B2 (Fig. 5b, *light blue*, and 5e). Instead, the side chain of R120 in CYP11B2 is oriented upward and interacts with the main chain carbonyl oxygen of W116 (Fig. 5b, *light blue*), creating more space in the innermost area (Fig. 5e). Second, the side chain of W116 in the CYP11B1 mutant is located at a lower and left-hand position compared to that in the wild type (Fig. 5a, *orange vs* *light gray*), whereas the side chain of W116 in DOC-bound CYP11B2 is located at a higher and right-hand position (Fig. 5b, *light blue*). The distance from the C3 carbonyl oxygen atom (O3) of DOC to the Cζ3 atom (CZ3) of the side chain of W116 is 4.1 ​Å in DOC-bound CYP11B2 (Fig. 5b, *light blue*). When DOC is placed into the substrate-binding site of the mutant and the wild type of CYP11B1 at the same position relative to the heme in the CYP11B2 structure, the distances from O3 of DOC to CZ3 of W116 are 2.0 ​Å and 2.8 ​Å, respectively. These distance estimations indicate that the innermost area in the substrate-binding site of CYP11B1 could cause steric hindrance more severely in the mutant than in the wild type with DOC which occupied the same position as in the crystal structure of CYP11B2.

To further examine whether the substrate-binding sites are connected to bulk water through a water channel other than the substrate access channel, molecular surfaces were calculated with a probe radius of 1.4 ​Å. Both mutant and wild type of CYP11B1 do not have an additional channel for passage of a water molecule (Fig. 4e and f). In contrast, DOC-bound CYP11B2 has a water channel, above helix I, extending from the space around O3 of DOC to bulk water as reported previously (Strushkevich et al., 2013), while the channel is closed in (*R*)-fadrozole-bound CYP11B2 (Fig. 4g and h). Since the channel is too narrow for steroid molecules, it is unlikely to function as an egress route for a product, as discussed later. In CYP11B1, mainly due to the downward shift of helix G, the side chains of W116, R120, W260, F264, E310, and A313 are shifted as they block formation of the channel more strongly in the mutant than in the wild type (Figs. S8a and b). In CYP11B2, the side chains of the same residues form the space for the channel (Fig. S8c). Thus, the enlarged space found in the substrate-binding site of DOC-bound CYP11B2 is accessible to bulk water. This is consistent with the MD simulation of DOC-bound CYP11B2 where water molecules form a hydrogen bonding network for the substrate binding, as described below.

From the comparisons of the substrate-binding sites among the three crystal structures in combination with their catalytic activities of 11β,18-dihydroxylation, the spatial dimensions of the innermost part of the sites correlate with the dihydroxylase activities.

### 2.4 MD simulation reveals structural differences in the substrate-binding sites

**Fig. 6:**
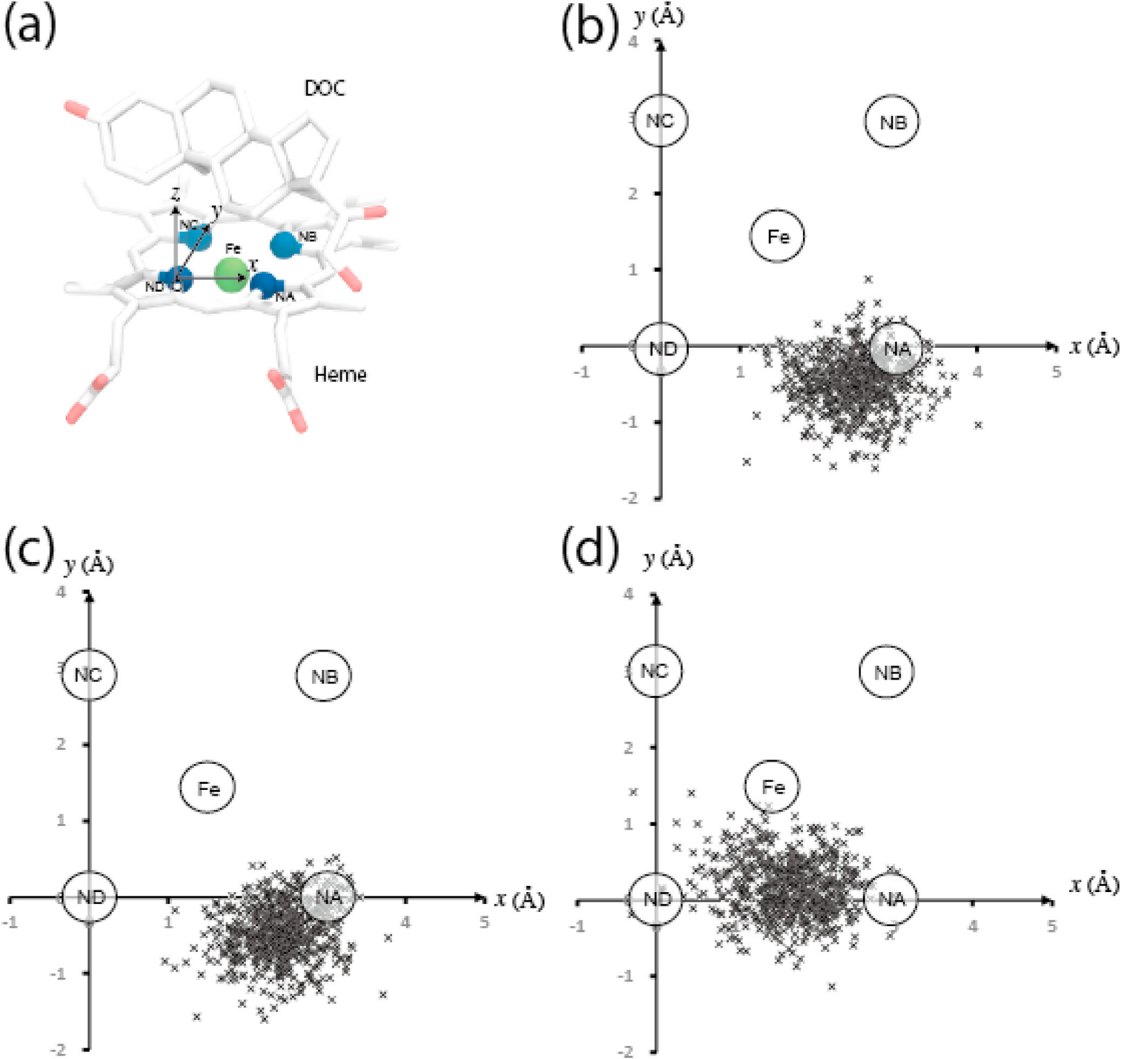
Projection of geometric center of DOC onto heme plane in MD simulation. (a) Cartesian coordinate axes for projection of the geometric center of DOC. Nitrogen atom ND of porphyrin is placed at the coordinate origin. The positive direction of x-axis is the direction of the position vector of NA. The positive direction of z-axis is the direction of the cross product of a unit vector along x-axis and the position vector of NC, and the positive direction of y-axis is the direction of the cross vector of the unit vectors along x-axis and z-axis. (b–d) Scatterplots of the coordinates (x,y) of the geometric center of DOC calculated every 100 psec from total MD trajectories (60 nsec). (b) DOC-bound CYP11B1 mutant. (b) DOC-bound CYP11B1 wild type. (d) DOC-bound CYP11B2.

Crystal structures of substrate-bound forms of the CYP11B1 mutant and the wild type have not been solved. The structural differences described in the previous section were obtained from the comparisons among the crystal structures of the three enzymes each bound to the different ligands. We therefore carried out MD simulations to generate their DOC-bound forms, as the enzymes bound to the same ligand, using the crystal data. DOC-bound CYP11B2 was also generated by MD simulation using its own crystal data under the same conditions. First, we examined positions of the geometric centers of DOC in the MD trajectories by projecting them onto the heme plane (Fig. 6). The geometric centers observed in both CYP11B1 were distributed similarly to each other in the vicinity of the nitrogen atom NA (see Fig. 6a) of heme, whereas those in CYP11B2 were distributed with a shift by approx. 1 ​Å toward the heme iron and the nitrogen atom ND (Fig. 6b–d). These results showed that the geometric centers of DOC in both CYP11B1 are located more distant from the innermost area of the substrate-binding site compared to simulated CYP11B2. Average spatial distances from the heme iron to C11 of DOC were 4.8 ​Å and 4.9 ​Å in the mutant and the wild type, respectively, while that in CYP11B2 was 4.6 ​Å (Table 3), indicating that these distances did not markedly differ among the three enzymes. Average spatial distances from the heme iron to C18 in both CYP11B1 were 5.8 ​Å and 5.7 ​Å in the mutant and the wild type, respectively, which were longer than 5.0 ​Å of that in CYP11B2 by approx. 0.8 ​Å (Table 3), indicating that the distances from the heme iron to C18 in both CYP11B1 were much longer than that in CYP11B2.

**Table 3:** MD calculation of atomic distances (Å).


	Mutant CYP11B1		Wild type CYP11B1		CYP11B2	
	DOC	PDB (7E7F) Metyrapone	DOC	PDB (6M7X) (*S*)-Fadrozole	DOC	PDB (4DVQ) DOC
HEME FE-DOC C11	4.8 ​± ​0.3		4.9 ​± ​0.3		4.6 ​± ​0.3	4.2
HEME FE-DOC C18	5.8 ​± ​0.4		5.7 ​± ​0.3		5.0 ​± ​0.4	5.0

R120 NH1-E310 OE1	5.0 ​± ​0.1	4.8	3.2 ​± ​0.7	5.0	5.3 ​± ​1.3	6.3
R120 NH1-E310 OE2	2.8 ​± ​0.1	2.7	3.5 ​± ​0.8	2.9	6.5 ​± ​1.4	4.6
R120 NH2-E310 OE1	4.8 ​± ​0.1	5.1	3.6 ​± ​0.7	5.3	6.4 ​± ​1.6	8.4
R120 NH2-E310 OE2	2.9 ​± ​0.2	3.3	3.4 ​± ​0.8	3.6	7.4 ​± ​1.7	6.5

Data are presented as mean ​± ​standard deviation.

Second, analyses of average distances between the nitrogen atoms of R120 side chain and the oxygen atoms of E310 side chain indicated that ionic bonds were formed between them in DOC-bound mutant and wild-type CYP11B1 but not in CYP11B2 (Table 3). The standard deviations of the average distances suggest that the ionic bond in the CYP11B1 mutant is more stable than that in the wild type. These findings suggest that the side chains of R120 and E310 form as it were a wall in the innermost area of the substrate-binding site to reduce the space in CYP11B1, whereas the absence of such an ionic bond in CYP11B2 expands the space for the substrate binding.

Subsequently, amino acid residues close to DOC were analyzed by monitoring the residue atoms (except hydrogen) located at distance of ≤4.0 ​Å from carbon or oxygen atoms of DOC in the MD trajectories. The residues W116, F130, E310, A313, G314, G379, F381, L382, and F478 of the three enzymes were found to meet the conditions with occupancies ≥0.5 (Figs. S9a–c, *sticks*). Similarly, analysis of occupancies of the residue atoms located at distance of ≤4.0 ​Å from O3 of DOC revealed that the side chain atoms of W116 and F130 and the main chain atoms of A313 and G314 were closer to O3 of DOC in the CYP11B1 mutant than in the wild type but more distant in CYP11B2 (Fig. S9d and see also Figs. S9a–c, *balls*). Thus, the space around the C3 side of DOC is more restricted in the mutant than in the wild type of CYP11B1, whereas it is enlarged in CYP11B2 compared to those in both CYP11B1.

**Fig. 7:**
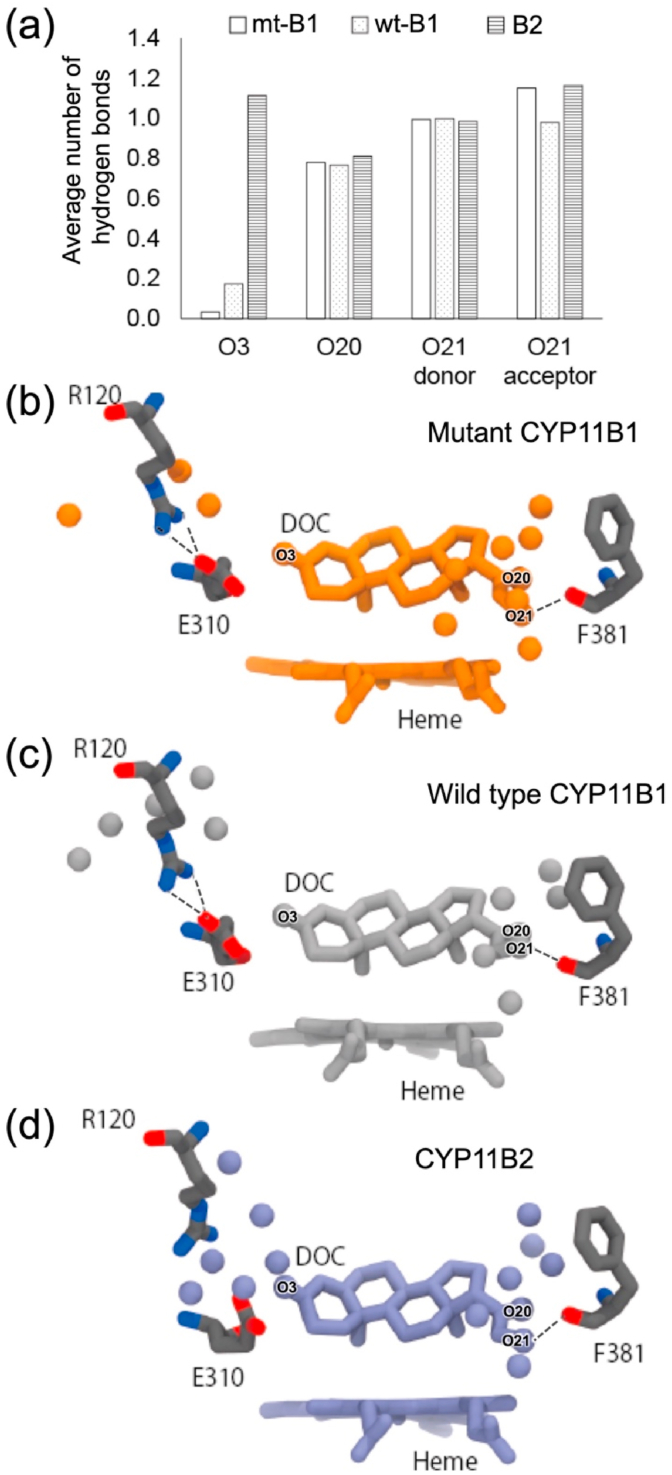
Hydrogen bond formed with oxygen atoms of DOC in MD simulation. (a) Average numbers of hydrogen bond formed with O3, O20, and O21 atoms of DOC in MD simulations of DOC-bound mutant CYP11B1 (mt-B1), wild type CYP11B1 (wt-B1), and CYP11B2 (B2). Representative MD snapshots of DOC-bound forms of (b) mutant CYP11B1, (c) wild type CYP11B1, and (d) CYP11B2 are shown. Oxygen atoms of water molecules located at a distance ≤5.0 ​Å from either oxygen atoms of DOC or side chain atoms of R120 or E310 are shown as balls. Oxygen atoms (O3, O20, and O21) of DOC are labeled. Ionic bonds formed between the side chains of R120 and E310 in panels (b) and (c) and hydrogen bond interactions between O21 and carbonyl oxygen of F381 in panels (b)–(d) are indicated by dashed lines.

To further explore the positioning of DOC in the substrate-binding site of the simulated complexes, formation of hydrogen bond involving the three oxygen atoms, O3, C20 carbonyl oxygen (O20), and C21 hydroxyl oxygen (O21), of DOC was examined. O3 of DOC hardly formed hydrogen bonds in the mutant of CYP11B1 (average number of 0.03) and rarely in the wild type (average number of 0.16), whereas O3 of DOC in CYP11B2 formed hydrogen bonds to water molecules with average number of 1.1 (Fig. 7a). In the representative MD snapshots, water molecules were not observed in the vicinity of O3 in the CYP11B1 mutant and the wild type (Fig. 7b and c), whereas they were observed in CYP11B2 between O3 of DOC and the side chains of R120 and E310, forming a hydrogen bonding network (Fig. 7d). In the reported crystal structure of DOC-bound CYP11B2 determined at 2.5 ​Å resolution such water molecules are not detected (Strushkevich et al., 2013). On the other hand, formation of hydrogen bonds involving O20 and O21 of DOC did not differ markedly among the three enzymes (Fig. 7a). O20 accepted hydrogen bonds from water molecules (average numbers of approx. 0.8), and O21 donated hydrogen bond to carbonyl oxygen of F381 (approx. 1.0) and accepted from water molecules (approx. 1.0 to 1.2) (Fig. 7a). The key difference in the substrate-binding sites between CYP11B1 and CYP11B2 is that very few water molecules form hydrogen bonds with O3 in CYP11B1, especially in the mutant, whereas a hydrogen bonding network involving water molecules is formed with O3 and the side chains of R120 and E310 in CYP11B2.

In addition to the DOC-bound forms, we performed MD calculations to generate metyrapone-bound forms of the CYP11B1 mutant and the wild type, as another set of the enzymes bound to the same ligand. In comparison of the spatial dimensions of the substrate-binding sites, the distance distributions of W116 CZ3-F130 CG were clearly different between the mutant and the wild type (Fig. S10a). The average value for the mutant (4.4 ​Å) was lower by 0.3 ​Å than that for the wile type (4.7 ​Å). This is in agreement with the corresponding value of 0.4 ​Å obtained from the crystal structures (4.0 ​Å in the metyrapone-bound mutant and 4.4 ​Å in the (*S*)-fadrozole-bound wild type). Regarding the ionic bond between the side chains of R120 and E310, the distance distributions of R120 NH-E310 OE exhibited different patterns in peak shapes between the two enzymes (Fig. S10b). The shortest average distance among the four distributions in the mutant (2.8 ​Å; Fig. S10b upper panel, blue) was smaller by 0.3 ​Å than that in the wild type (3.1 ​Å; Fig. S10b lower panel, blue). This is also consistent with the corresponding value of 0.2 ​Å from the crystallographic results (2.7 ​Å in the mutant bound to metyrapone and 2.9 ​Å in the wild type bound to (*S*)-fadrozole). The results coupled with those from the simulations of the DOC-bound forms provided strong evidence indicating that the spatial restriction in the substrate-binding site of the mutant was caused by the amino acid replacements.

## 3 Discussion

The present study shows that the functional divergence of the CYP11B enzymes in the ability to catalyze 18-hydroxylation following 11β-hydroxylation depends on spatial dimension in the substrate-binding site. The finding is based on structural analyses of the CYP11B1 mutant deficient in the 11β,18-dihydroxylase activity but retaining 11β- and 18-monohydroxylation activities of the wild type. The correlation between the dihydroxylase activity and the spatial dimension was derived from comparison of the present results with the previous data including the crystal structures of the CYP11B1 wild type and CYP11B2 (Brixius-Anderko and Scott, 2019; Strushkevich et al., 2013). MD simulations of DOC-bound forms of the three enzymes corroborated the correlation and indicated that the additional space of CYP11B2 had a hydrogen bonding network involving water molecules that position DOC in the substrate-binding site. This study reveals the structural differences in the substrate-binding site between CYP11B1 and CYP11B2 and advances understanding of the molecular mechanisms for the differential production of glucocorticoid and mineralocorticoid.

Because the CYP11B1 wild type is able to catalyze the 11β,18-dihydroxylation, a step necessary for aldosterone synthesis, the enzyme has in part a functional feature of CYP11B2. The deficiency in the dihydroxylase activity of the CYP11B1 mutant generated in the present study means that the mutant is more distantly related to CYP11B2 in terms of catalytic ability. Employing the CYP11B1 mutant, the present study highlights the structural differences in the substrate-binding site between CYP11B1 and CYP11B2. The mutant of CYP11B1 serves as a molecular entity that enlarged the differences in both structure and function between CYP11B1 and CYP11B2.

***Spatial requirement for successive hydroxylation*** Assuming that a single enzyme catalyzes hydroxylation of C18 in addition to C11β successively, the substrate-binding site has to position C18 of 11β-hydroxy steroid within the distance of hydroxylation by heme-bound active oxygen. This implies that the space around the C3 side of steroid occupying the substrate-binding site has a key role for the successive catalytic activity. The present study indicates two major structural elements to characterize the innermost area of the substrate-binding site. As described, one is ionic interaction between the side chains of R120 and E310, and the other is arrangement of the side chain of W116. In CYP11B1, formation of the ionic bond between R120 and E310 and a lower position of W116 side chain are likely to induce steric hindrance with the C3 side of 11β-hydroxy steroid when it is placed so that C18 is hydroxylated. In CYP11B2, additional space due to the absence of the ionic bond and higher position of W116 side chain avoids such steric hindrance and allows for successive 18-hydroxylation more efficiently.

The putative water channel connecting to the innermost space in the substrate-binding site of CYP11B2 (Fig. 4g) was previously assumed to be an egress route for 11β-hydroxylated product of both CYP11B enzymes (Strushkevich et al., 2013). However, the absence of the channel in the CYP11B1 mutant of the present study and in the wild type excludes such a functional role as an egress route (Fig. 4e and f). One may ask what roles the water channel plays in the catalysis of CYP11B2. The channel can deliver water molecules that form the hydrogen bonding network with the side chains of R120 and E310 and O3 of DOC. The hydration of the side chains of R120 and E310 stabilizes the charges and generates the additional space. Besides, the water molecules position DOC via the hydrogen bonding to O3 of steroid. When the water molecules are replaced by O3 which directly forms a hydrogen bond with the R120 side chain in the successive catalysis, such a rearrangement of hydrogen bond formation shifts the steroid molecule toward the innermost area to ensure that C18 is readily located closer to the heme iron. In the opposite side of the substrate-binding site, O20/O21 of steroid potentially interacts with the carbonyl oxygen of F381 through added water molecules (Fig. 7d). The positional change of steroid molecule in the substrate-binding site in parallel with such a rearrangement of hydrogen bond formation could be a possible mechanism to facilitate the successive 18-hydroxylations of 11β-hydroxy steroid to synthesize aldosterone in CYP11B2.

***Functional alteration induced by amino acid substitutions outside of the substrate-binding site*** Previous studies have examined catalytic activities of the human CYP11B mutants carrying an exchange(s) of the isoform-specific amino acid residues between CYP11B1 and CYP11B2 to identify residues responsible for the catalytic differences (Bottner et al., 1996, 1998; Curnow et al., 1997; Pascoe et al., 1992a). Based on the reported results and the present results obtained with the novel mutant, these mutants can be classified into four groups (Table S1). (i) The CYP11B1 mutant generated in this study has only monohydroxylase activities of primary 11β-hydroxylation and secondary 18-hydroxylation. (ii) CYP11B2 G288S has a low level of 11β,18-dihydroxylase activity similar to that of the wild type of CYP11B1 (Curnow et al., 1997). (iii) CYP11B1 S288G has 11β,18-dihydroxylase activity, which is higher than that of wild type CYP11B1 and comparable to that of wild type CYP11B2, but no aldosterone synthase activity (Curnow et al., 1997). The activities of CYP11B1 S288G are in between those of the two wild type enzymes. (iv) CYP11B1 S288G/V320A gains aldosterone synthase activity as well as 11β,18-dihydroxylase activity that are comparable to those of wild type CYP11B2 (Curnow et al., 1997). Thus, S288 and V320 diminish the successive 18-hydroxylation, while G288 and A320 enhance the successive 18-hydroxylation. This indicates that these residues could dictate the isoform-specific structures for positioning of substrate and/or intermediate product in their substrate-binding sites. A CYP11B2 mutant carrying R181W/V386A, which was identified in patients with congenital aldosterone synthase deficiency (Pascoe et al., 1992b), exhibits catalytic activities corresponding to the group (iii) (Table S1). Another mutant CYP11B2 T185I, which also causes aldosterone synthase deficiency (Peter et al., 1998), is likely to be classified into this group. Notably, all of the amino acid replacements are located outside their substrate-binding site. The previous studies, however, including those performing molecular modeling or docking studies on the CYP11B enzymes (Hobler et al., 2012; Roumen et al., 2007) could not propose a putative scheme that illustrates how the isoform-specific amino acid residues dictate distinct structural elements in their substrate-binding sites for the catalytic divergence.

***Roles of the isoform-specific residues to form the distinct structures*** Since the present study reveals that the structural difference in the innermost area of the substrate-binding sites between CYP11B1 and CYP1B2 dictates the functional difference, we suggest possible roles of the isoform-specific amino acid residues at 288 and 320 to form the distinct structures to differentiate their catalytic activities (Fig. S11). S288 of CYP11B1 is located on the N-terminus of helix H in proximity to the C-terminus of helix E. The side chain of S288 in CYP11B1 interacts with the amide nitrogen of A291 by hydrogen bonding and contributes to the shifting of helix H outward from the heme (Fig. S11a), while G288 in CYP11B2 does not have such a structural effect on helix H (Fig. S11c). The shift of helix H in CYP11B1 is accompanied by the characteristic shift of helix G (Fig. 3b right) due to hydrophobic interactions between the side chains including those of V290 and L294 on helix H and I271 and I274 on helix G. V320 on helix I of CYP11B1 is located behind the substrate-binding site (Fig. S11b). Compared to A320 in CYP11B2, the larger side chain of V320 possibly changes the side chain orientations of F193 and I197 on helix E and then leads to distortion of helical structure in the middle part of helix I including V316 and a shift of the side chain of A313 (Fig. S11d). The resulting structural shifts of the side chains of the residues such as W260 and F264 (helix G) and A313 (helix I) in CYP11B1 work in an integrated fashion to block formation of the water channel (Figs. S8a and b) and reduce the innermost space of the substrate-binding site (Fig. 5c and d).

Besides the findings obtained with the CYP11B1 mutant, the present work describes the biochemical properties of the wild type of CYP11B1 in detail including spectroscopic properties, affinities for steroids and ligands, and enzyme kinetics. It is of significance that catalytic activities of 11β- and 18-monohydroxylations and 11β,18-dihydroxylations were determined under the steady-state conditions. In particular, the present observation confirms low-spin state of the heme iron in the substrate-free ferric form of wild type CYP11B1. This is consistent with model cytochrome P450cam from *Pseudomonas putida*, where substrate-free ferric form is in ferric low-spin state. The present result makes a correction to the previous report showing the high-spin state of purified CYP11B1 in the absence of substrates (Zöllner et al., 2008). Although the exact reason for the discrepancy is unknown, this could be caused by the presence of a chemical, which changes the spin state of the heme iron of substrate-free CYP11B1 from low to high by interaction in a similar manner to that of a substrate, such as Cymal-5 (Figs. S4e and f). Another thing to note is that as demonstrated the substrate-bound CYP11B1 both of the wild type and the mutant were partially reduced by dithionite. The difficulty in reduction of DOC-bound ferric CYP11B1 in high-spin state by dithionite could be due to a very low redox potential. Since similar observations were previously reported for purified CYP11B1 (Zöllner et al., 2008) and CYP11B2 (Hobler et al., 2012), these phenomena are common to the CYP11B proteins. Finally, it is important to note that the present findings on the substrate-binding sites of the CYP11B enzymes can provide strategic insights into drug design to distinguish between the two enzymes. Further analyses to address the structures of substrate-bound CYP11B1 and intermediate product-bound CYP11B2 are indispensable.

## 4 Materials and Methods

### 4.1 Expression and purification of human CYP11B1

Two forms, a wild type and a mutant, of recombinant human CYP11B1 were expressed as 486-amino acid residue polypeptide (Fig. S2). They had modifications at amino and carboxy termini of the mature form 479-residue polypeptide (corresponding to the residue numbers 25–503) that is derived by cleavage of the amino-terminal 24-residue fragment (Ogishima et al., 1989) from the 503-residue precursor (GenBank accession number NM_000497). The three residues at the amino-terminus (GTR: residue numbers 25–27) of the mature enzyme were replaced with MATK, and the original carboxy-terminus at residue number 503 was extended with addition of hexahistidine residues. The mutant carried the replacement at six amino acid residues (W49R, L50N, L53N, W56R, L244N, and W247N; see Fig. S2, residues in red), where the wild type enzyme possibly interacts with the inner mitochondrial membrane. DNA fragments encoding the two forms were cloned into pET17b vector (Novagen) utilizing *Nde*I and *Hin*dIII restriction sites. The nucleotide sequences of the two constructs were confirmed by the dideoxy sequencing. *E. coli* BL21(DE3) carrying pGro12 was transformed with the pET17b vectors carrying the CYP11B1 constructs.

Recombinant proteins were expressed and purified in similar manners as described previously (Hobler et al., 2012; Zöllner et al., 2008). For induction, cultures were incubated at 28 ​°C for 27 or 45 ​h in a rotary shaker at a rate of 200 ​rpm. Cells were collected and resuspended with a buffer containing 50 ​mM Tris-HCl (pH 7.5), 250 ​mM sucrose, 0.5 ​mM Na EDTA, and 0.5 ​mg/ml lysozyme. Proteins were solubilized from the cells by sonication with a buffer consisting of 50 ​mM potassium phosphate (pH 7.4), 500 ​mM sodium acetate, 20% (v/v) glycerol, 0.1 ​mM EDTA, 0.1 ​mM DTT, 1.5% (w/v) sodium cholate (Sigma), 1.5% (v/v) Tween 20 (Sigma), 0.1 ​mM PMSF. Purification by column chromatography was carried out using Ni-NTA Superflow (Qiagen), DEAE Sepharose FF, and SP Sepharose FF (GE Healthcare). CYP11B1 proteins were eluted from SP Sepharose columns with a buffer consisting of 40 ​mM potassium phosphate (pH 7.4), 20% glycerol, 10 ​mM imidazole (Nacalai Tesque), 1% sodium cholate (Dojindo Molecular Technologies), 0.1% Tween 20, 0.1 ​mM PMSF, 0.1 ​mM Na EDTA, 0.1 ​mM DTT, and 75 ​mM NaCl for the wild type or 120 ​mM NaCl for the mutant. The eluates were analyzed using SDS-polyacrylamide gel (Extra PAGE One Precast Gel 7.5–15%, Nacalai Tesque) with molecular weight markers (LMW marker kit, GE Healthcare), and proteins were stained with Coomassie Brilliant blue. CYP11B1 proteins (50 ​kDa) were apparently homogeneous except for very faint bands at 25 and 28 ​kDa (Fig. S3). Concentrations of CYP11B1 were determined spectroscopically using the molecular extinction coefficient *ε*_393_ ​= ​96 ​mM^−1^ ​cm^−1^ for high-spin state induced by binding of substrate to cytochromes P450 (Hashimoto-Yutsudo et al., 1980) because difference spectra of reduced CO complexes of CYP11B1 gave underestimation due to limited reduction by sodium dithionite and instability of the reduced CO complexes, as described earlier sections (Hobler et al., 2012; Zöllner et al., 2008).

### 4.2 Preparation of bovine adrenodoxin and NADPH-adrenodoxin oxidoreductase

cDNA encoding bovine adrenodoxin was kindly provided by Dr. Yasuhiro Sagara (Sagara et al., 1992) and used as a template for PCR. The initiation methionine codon was added to the DNA fragment encoding the adrenodoxin polypeptide (residues from 60 to 182 of the 186-residue precursor), and a hexahistidine tag sequence was added at the carboxy terminus. The resulting DNA fragment encoding 130-amino acid residue polypeptide (MSSED … MGMNSH_6_) was cloned into pET17b utilizing *Nde*I and *Hin*dIII restriction sites. Expression and extraction of the recombinant bovine adrenodoxin was carried out as described above for CYP11B1 except that sodium cholate and Tween 20 were omitted. Homogeneous adrenodoxin (A_414_/A_280_ ​= ​0.86–0.90 (Suhara et al., 1972b)) was obtained using an Ni-NTA column. Bovine NADPH-adrenodoxin oxidoreductase was a kind gift provided by Dr. Fumiko Mitani, and it was prepared from bovine adrenal glands with the methods described previously (Suhara et al., 1972a). Concentrations of oxidized forms of adrenodoxin and adrenodoxin reductase were determined spectroscopically using the molecular extinction coefficients of *ε*_414_ ​= ​11.0 mM^−1^cm^−1^ (Huang and Kimura, 1973) and *ε*_450_ ​= ​10.9 mM^−1^cm^−1^ (Chu and Kimura, 1973), respectively.

### 4.3 Spectroscopy

Spectroscopic studies were carried out at room temperature using a Jasco UV–visible spectrophotometer V-530. Purified enzymes were diluted to 1.0 ​μM for the wild type and 0.8–1.2 ​μM for the mutant with the buffer consisting of 50 ​mM potassium phosphate (pH 7.4), 150 ​mM NaCl, 20% (v/v) glycerol, and 0.05% (v/v) Tween 20. Substrate DOC (Sigma) at 400 ​μM, sodium dithionite, and CO were added sequentially, and absolute spectra were recorded after each step. Cymal-5, unlike other several detergents we used with the enzyme preparations, was found to induce high-spin state of the heme iron and to give higher levels of reduced CO forms of both wild-type and mutant than DOC. The data obtained with 5 ​mM Cymal-5 (Anatrace) in place of the substrate were also included in Fig. S4. Dissociation constants for steroids and ligands were determined from spectral changes caused by titration with increasing concentrations of them. Data were analyzed using non-linear regression (Prism 8, GraphPad Software) to calculate *K*_d_ values. Titration with metyrapone (Sigma) was carried out in the presence of DOC at 320 ​μM as a competitor in order to evaluate the *K*_d_ values smaller than the concentrations of CYP11B1. The dissociation constants were calculated from the apparent values by using the *K*_d_ values for DOC as the inhibitory constants. To exclude the possibility that binding of cortisol or corticosterone holds the heme iron in low-spin state, effects of 1 ​mM cortisol (Sigma) or corticosterone (Sigma) as a competitor on the *K*_d_ values for DOC or DOF (Sigma) were tested. The steroids and ligands used for spectroscopic analyses were dissolved in dimethyl sulfoxide.

### 4.4 Enzyme activity

Catalytic activities of the purified CYP111B1 enzymes were examined using a reconstituted system including an NADPH-regenerating system, adrenodoxin reductase, and adrenodoxin as described previously (Hobler et al., 2012; Zöllner et al., 2008). Assay mixture (0.2 ​mL) consisted of 50 ​mM Hepes (pH 7.5), 5 ​mM MgCl_2_ 0.1 ​mM EDTA, 20% (v/v) glycerol, 5 ​mM glucose-6-phosphate, 2 U/ml glucose-6-phosphate dehydrogenase (Roche), 1 ​mM NADPH (Roche), 0.125 ​μM bovine adrenodoxin reductase, 5 ​μM bovine adrenodoxin, 2 ​nM CYP11B1, 100 ​μM 1,2-dilauroyl-*sn*-glycero-3-phosphocholine (DLPC, Sigma), and 0–80 ​μM DOF or DOC. Products converted from the substrates DOF or DOC were analyzed by liquid chromatography-tandem mass spectrometry. Increases in concentrations of adrenodoxin reductase from 0.125 ​μM or of adrenodoxin from 5 ​μM did not raise turnover numbers of both CYP11B1 enzymes. Steroids and DLPC used for activity assays were prepared by dissolving in ethanol. Reaction was started with addition of NADPH, incubated at 37 ​°C for 5 ​min, and stopped by addition of 1 ​mL chloroform followed by vortexing. Progesterone (Sigma) at 500 ​nM was included in the reaction mixture as internal standard. Steroids were extracted from the reaction mixtures once with chloroform and evaporated and were dissolved with 50 ​μL of 50% (v/v) methanol. Aliquots (5 ​μL) were analyzed using an Nexera UHPLC system coupled with LCMS-8030plus triple quadrupole mass spectrometer (Shimadzu). A C18 column (ACQUITY CSH™ 1.7 ​μm, 2.1 ​× ​150 ​mm, Waters) was used, and the temperature of the column oven was kept at 40 ​°C. Running solvents used for the chromatography were: (A) H_2_O ​+ ​0.05% (v/v) formic acid and (B) acetonitrile ​+ ​0.05% (v/v) formic acid. The following gradients were applied: 0–2.0 ​min, 5% B; 2.0–3.0 ​min, 5–30% B; 3.0–12.0 ​min, 30–98% B; 12.0–18.0 ​min, 98% B; 18.0–18.5 ​min, 98-5% B; 18.5–24.0 ​min, 5% B at flow rate of 0.2 ​ml/min. MS/MS was carried out with electrospray ionization at positive ion mode, and multiple reaction monitoring transition are outlined in Table S2. DOF, cortisol, 18-hydroxy cortisol, DOC, corticosterone, 18-hydroxy DOC, 18-hydroxy corticosterone, and aldosterone were quantitated by correlating their peak areas with those of known standards and by referring recovery rates of the internal standard. Steroid standards were obtained from Sigma except for 18-hydroxy cortisol (Steraloids). 18-hydroxy DOF and 18-oxo-cortisol were unavailable. Data were analyzed using non-linear regression (Prism 8) to calculate *k*_cat_ and *K*_m_ values.

### 4.5 Crystallization

For crystallization, imidazole bound to the purified CYP11B1 mutant was replaced with metyrapone using ultrafiltration devices (Amicon Ultra-15 30K, Millipore and Vivaspin 6 50K, GE Healthcare) by three cycles of dilution and concentration with a buffer consisting of 20 ​mM potassium phosphate (pH 7.4), 20% (v/v) glycerol, 150 ​mM NaCl, 0.05% (w/v) Fos-choline 12 (Anatrace), 0.1 ​mM Na EDTA, 0.1 ​mM DTT, and 100 ​μM metyrapone. Crystals were grown in drops prepared by mixing of 1 ​μL of 500 ​μM enzyme with 1 ​μL of a precipitant consisting of 100 ​mM potassium phosphate (pH 7.4), 150 ​mM NaCl, and 4% (w/v) PEG 3350 (Hampton Research) on sitting drop plates at 4 ​°C, and the reservoir solution used was 20% v/v glycerol.

### 4.6 Data collection and structure determination

Crystals were cryoprotected by soaking in increasing concentrations of glycerol from 20% to 25% and then flash-frozen in liquid nitrogen. X-ray diffraction data were collected using a wavelength of 1.0 ​Å ​at BL41XU in SPring-8 and processed using XDS (Kabsch, 2010). Data collection statistics are shown in Table 2. The initial phase of the CYP11B1 crystal was obtained using the molecular replacement (MR) method using the program Phaser (McCoy et al., 2007). The structure of CYP11B2 (PDB ID: 4DVQ) was used as the search model for MR. The protein model of MR solution was manually rebuilt using the program Coot (Emsley and Cowtan, 2004). The atomic coordinates were further refined with multiple rounds of manual rebuilding followed by restrained refinement using Phenix (Adams et al., 2010). The geometries of the model were evaluated by MolProbity (Davis et al., 2007). Ramachandran plot analysis of the main chain showed that 97% were in favored regions. Rotamer analysis of the side chain (chi1-chi2 plot) showed that 98% of residues lie in the favored regions. Figures showing the crystal structures are produced with PyMOL (DeLano, 2019).

### 4.7 MD simulation

The structural models of CYP11B1 and CYP11B2 were constructed from the crystal structures of the metyrapone-bound CYP11B1 mutant in the present study, (*S*)-fadrozole-bound wild type CYP11B1 (PDB ID: 6M7X), and DOC-bound CYP11B2 (PDB ID: 4DVQ). The missing *N*- and C-terminal residues with free amino and carboxyl groups at the termini were modeled by the LEaP program in AmberTools (Case et al., 2018), while the missing residues 281–286 in the wild type CYP11B1 and 432–437 in CYP11B2 were modeled using the crystal structure of the CYP11B1 mutant in which the atomic coordinates of these residues have been identified. All crystallographic water molecules in the X-ray structures were included in the systems, while all cholate and glycerol molecules in the CYP11B1 mutant and sulfate ions in CYP11B2 were excluded. The protonation states of the proteins were examined by the H++ server (Anandakrishnan et al., 2012). Each protein was then solvated in a rectangular box of TIP3P water molecules under periodic boundary conditions, followed by neutralization by the addition of Cl^−^ ions.

The force field for the nonstandard residues, i.e., heme, proximal C450, nitrogen coordinating ligands (metyrapone and (*S*)-fadrozole), and DOC, was built by the antechamber and MCPB.py in AmberTools18 (Li and Merz, 2016), while the Amber ff14SB force field was used for standard residues. The heme complexes were assumed to be in the oxidized form, and the nitrogen coordinating ligand-bound enzymes were in a six-coordinated low-spin state, whereas the DOC-bound enzymes were in a five-coordinated high-spin state. The heme complexes were described by the bonded model, where the porphyrin, the proximal cysteine residues, and an inhibitor were bound to the heme iron. All of the parameters of the heme complexes were derived from quantum mechanical calculations by density functional theory (B3LYP/6-31G∗) using Gaussian 16 (Frisch et al., 2016). The force constants were calculated from the method proposed by Seminario (1996), which uses sub-matrices of the Cartesian Hessian matrix obtained from the quantum mechanical calculations. For the partial charges, the restrained electrostatic potential (RESP) fitting scheme was used. The VDW radii for the RESP fits and Lennard-Jones parameters for Fe were taken from the work of (Li et al. (2015)), while those for the remaining porphyrin and ligands were adopted from the general AMBER force field (GAFF). The parameters of metyrapone, (*S*)-fadrozole, and DOC were determined by the AM1-BCC charge method with GAFF.

The initial geometries of the DOC complexes of the CYP11B1 mutant and the wild type and their metyrapone complexes were constructed from their ligand-bound CYP11B1 structures thermally equilibrated at 300 ​K and 1 ​atm by 20-ns MD simulations. After replacing the metyrapone or (*S*)-fadrozole molecules with DOC and equilibrating the systems, a 60-ns production run at 300 ​K and 1 ​atm was performed for the DOC complexes of the CYP11B1 mutant and the wild type, as well as for that of CYP11B2. For the metyrapone complexes of the CYP11B1mutant and the wild type, 10 ​100-ns runs at 300 ​K and 1 atom were performed using GROMACS 2019 (Abraham et al., 2019). The rest of the simulations was conducted using Amber18 (Case et al., 2018). The Berendsen weak-coupling algorithm and isotropic position scaling, the SHAKE method, and the particle-mesh Ewald method with a cutoff of 10 ​Å were used in the Amber simulations. Temperature coupling using velocity rescaling with a stochastic term and isotropic Berendsen exponential relaxation pressure coupling, the LINCS method, and the particle-mesh Ewald method with a cutoff of 10 ​Å were used in the GROMACS simulations. Figures derived from the MD simulations were produced by VMD (Humphrey et al., 1996).

## Accession number

The atomic coordinates and the structure factors of the metyrapone-bound mutant of CYP11B1 have been deposited in the Protein Data Bank under the accession code 7E7F.

## CRediT authorship contribution statement

**Kuniaki Mukai:** Conceptualization, Investigation, Visualization, Writing – original draft, Writing – review & editing. **Hiroshi Sugimoto:** Investigation, Formal analysis, Visualization, Writing – original draft, Writing – review & editing. **Katsumasa Kamiya:** Investigation, Formal analysis, Visualization, Writing – original draft, Writing – review & editing. **Reiko Suzuki:** Investigation, Visualization. **Tomomi Matsuura:** Investigation, Visualization. **Takako Hishiki:** Investigation, Visualization, Writing – original draft. **Hideo Shimada:** Conceptualization, Writing – review & editing. **Yoshitsugu Shiro:** Writing – review & editing. **Makoto Suematsu:** Writing – review & editing. **Norio Kagawa:** Writing – review & editing.

## Declaration of competing interest

The authors declare that they have no known competing financial interests or personal relationships that could have appeared to influence the work reported in this paper.
