# Supplementary material for: Spatially restricted substrate-binding site of cortisol-synthesizing CYP11B1 limits multiple hydroxylations and hinders aldosterone synthesis
Source: Curr Res Struct Biol. 2021 Aug 26;3:192–205. doi: 10.1016/j.crstbi.2021.08.001 (PMC8408562; doi:10.1016/j.crstbi.2021.08.001)
Supplement: Multimedia component 2 [file mmc2.pdf]

Supplementary information for

Spatially restricted substrate-binding site of cortisol-synthesizing CYP11B1 limits multiple hydroxylations and hinders aldosterone synthesis

Kuniaki Mukai*, Hiroshi Sugimoto, Katsumasa Kamiya*, Reiko Suzuki, Tomomi Matsuura, Takako Hishiki, Hideo Shimada, Yoshitsugu Shiro, Makoto Suematsu, and Norio Kagawa

*Corresponding authors: k-mukai@keio.jp (K. Mukai) and katsumasa.kamiya@gen.kanagawa-it.ac.jp (K. Kamiya).

Table S1. Classification of wild types and mutants of human CYP11B enzymes on the basis of catalytic activities.

		(i)	(ii)		(iii)	(iv)	
	CYP11B1	Mutant [a] (this study)	Wild type [a] (this study)	Wild type [b,c]	S288G [b]	S288G/ V320A [b]	
Activity	CYP11B2			G288S [b]	R181W/ V386A [c]	Wild type [b,c]	Wild type [d]
11β-OHase		95.8% (+++)	94.9% (+++)	+++	+++	+++	92.6% (+++)
18-OHase		4.2% (++)	4.7% (++)	nt [e]	nt [e]	nt [e]	4.2% (++)
11β,18-diOHase		0% (-)	0.4% (+)	+	++	++	2.7% (++)
11β,18-diOHase,18-oxidase		0% (-)	0% (-)	-	-	+	0.5% (+)

Relative activities in % are molar product ratios calculated from the k_{cat} values measured with substrate DOC for the CYP11B1 mutant and the wild type in this work and for CYP11B2 [1]. Percentages 0, >0 and ≤1, >1 and ≤10, and >10 are indicated by (-), (+), (++), and (+++), respectively. For comparison to the published data on known mutants [2, 3], amounts of steroids produced from DOC by mutant enzymes in transfection assays are estimated by comparing those produced by wild type enzymes of CYP11B1 and CYP11B2 used as controls, and the activities are indicated by -, +, ++, or +++ to ensure consistency with the data from the steady-state kinetic analyses using purified CYP11B1 in this study and CYP11B2 [1].

[a] From steady-state kinetics analyses in this study.

[b] From transfection experiments in reference [2].

[c] From transfection experiments in reference [3].

[d] From steady-state kinetics analyses in reference [1].

[e] nt, not tested.

References for Table S1

[1] Strushkevich N, Gilep AA, Shen L, Arrowsmith CH, Edwards AM, Usanov SA, et al. Structural insights into aldosterone synthase substrate specificity and targeted inhibition. Mol Endocrinol. 2013;27:315-24.

[2] Curnow KM, Mulatero P, Emeric-Blanchouin N, Aupetit-Faisant B, Corvol P, Pascoe L. The amino acid substitutions Ser288Gly and Val320Ala convert the cortisol producing enzyme, CYP11B1, into an aldosterone producing enzyme. Nat Struct Biol. 1997;4:32-5.

[3] Pascoe L, Curnow KM, Slutsker L, Rosler A, White PC. Mutations in the human CYP11B2 (aldosterone synthase) gene causing corticosterone methyloxidase II deficiency. Proc Natl Acad Sci U S A. 1992;89:4996-5000.

Table S2. Retention times, multiple reaction monitoring transitions, and detection parameters for MS/MS analysis.

Steroid	Retention time	Transition		MS/MS parameter		
		Precursor	Product	Q1	Collision energy	Q3
	(min)	(*m/z*)	(*m/z*)	(V)	(V)	(V)
Progesterone	11.714	315.25	109.00	-12	-26	-11
DOF	8.665	347.05	109.15	-25	-38	-17
Cortisol (F)	7.624	363.25	121.10	-11	-27	-21
18OH-Cortisol (18OH-F)	6.608	379.25	267.20	-18	-21	-18
DOC	9.786	331.25	108.95	-10	-30	-21
Corticosterone (B)	8.523	347.05	121.10	-13	-26	-12
18OH-DOC	8.009	347.25	97.00	-24	-24	-18
18OH-Corticosterone (18OH-B)	7.063	363.25	269.20	-26	-26	-28
Aldosterone	7.193	361.25	343.20	-18	-17	-17

DOF, 11-Deoxycortisol; DOC, 11-Deoxycorticosterone.

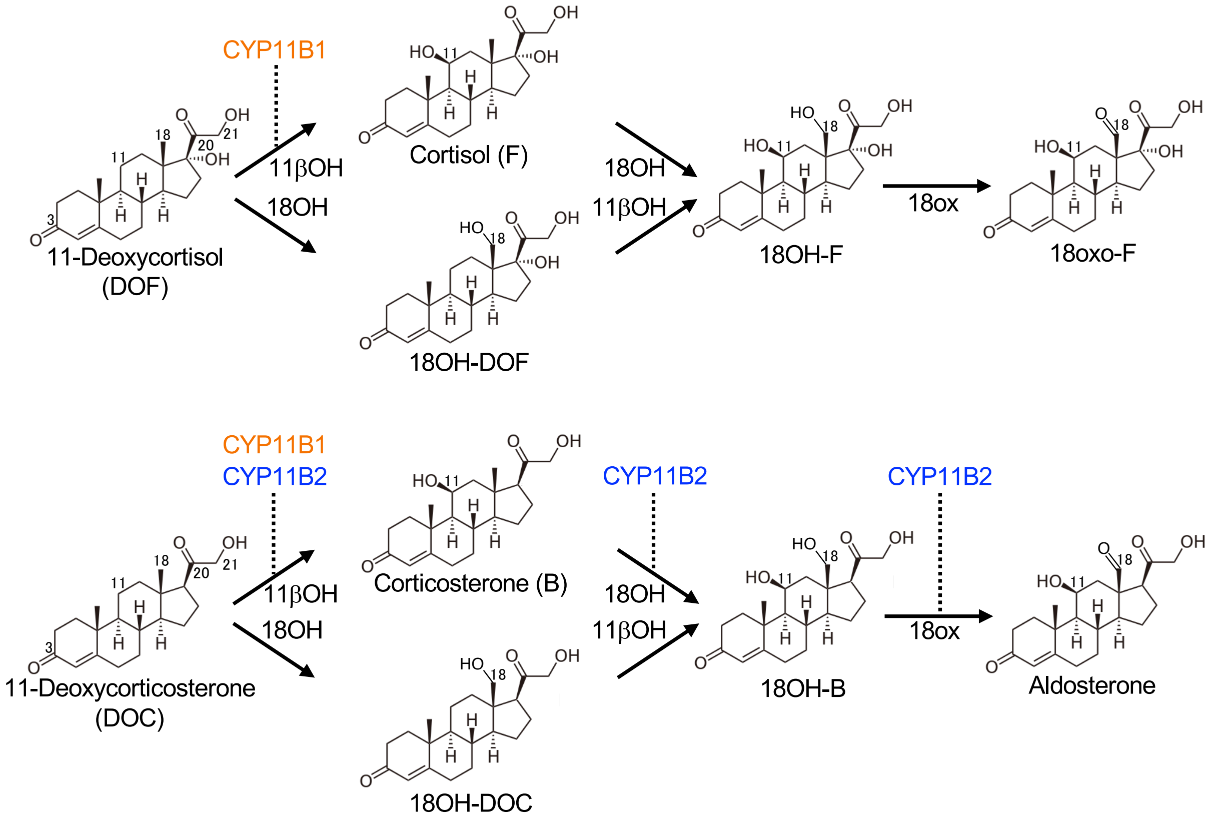

Figure S1. 11β-Hydroxylation, 18-hydroxylation, and 18-oxidation. 11-Deoxycortisol (DOF) and 11-deoxycorticosterone (DOC) are converted by 11β-hydroxylation (11βOH), 18-hydroxylation (18OH), and 18-oxidation (18ox). Dotted lines point reaction steps CYP11B1 or CYP11B2 catalyzes to play their physiological roles.

```
                                     10        20        30        40        50        60
Recombinant CYP11B1 mutant                                    MATKAARVPRTVLPFEAMPRRPGNRRNRLNQIRREQG
Recombinant CYP11B1 wild type                       .........................WL..L..W....
CYP11B1 precursor            MALRAKAEVCMAVPWLSLQRAQALG.R.....................WL..L..W....
CYP11B2 precursor            MALRAKAEVCVAAPWLSLQRARALG.R...A...........QH....WL..L..W....

                                     70        80        90       100       110       120
                             YEDLHLEVHQTFQELGPIFRYDLGGAGMVCVMLPEDVEKLQQVDSLHPHRMSLEPWVAYR
                             ............................................................
                             ............................................................
                             ..H....M.............N...PR.....................C..I........

                                    130       140       150       160       170       180
                             QHRGHKCGVFLLNGPEWRFNRLRLNPEVLSPNAVQRFLPMVDAVARDFSQALKKKVLQNA
                             ............................................................
                             ............................................................
                             ..........................D....K............................

                                    190       200       210       220       230       240
                             RGSLTLDVQPSIFHYTIEASNLALFGERLGLVGHSPSSASLNFLHALEVMFKSTVQLMFM
                             ............................................................
                             ............................................................
                             ............................................................

                                    250       260       270       280       290       300
                             PRSNSRNTSPKVWKEHFEAWDCIFQYGDNCIQKIYQELAFSRPQQYTSIVAELLLNAELS
                             ...L..W.....................................................
                             ...L..W.....................................................
                             ...L..WI................................N...H..G.......K....

                                    310       320       330       340       350       360
                             PDAIKANSMELTAGSVDTTVFPLLMTLFELARNPNVQQALRQESLAAAASISEHPQKATT
                             ............................................................
                             ............................................................
                             LE.................A..............D...I.....................

                                    370       380       390       400       410       420
                             ELPLLRAALKETLRLYPVGLFLERVASSDLVLQNYHIPAGTLVRVFLYSLGRNPALFPRP
                             ............................................................
                             ............................................................
                             .........................V.................Q.........A......

                                    430       440       450       460       470       480
                             ERYNPQRWLDIRGSGRNFYHVPFGFGMRQCLGRRLAEAEMLLLLHHVLKHLQVETLTQED
                             ............................................................
                             ............................................................
                             ..................H...............................FL........

                                    490       500
                             IKMVYSFILRPSMFPLLTFRAINHHHHHH                  486 residues
                             .............................                  486 residues
                             .......................                        503 residues
                             ...........GTS.........                        503 residues
```

Figure S2. Amino acid sequences of the recombinant CYP11B1 proteins and comparison to gene-encoded precursor polypeptides of CYP11B1 and CYP11B2. Amino acid sequences of the recombinant human CYP11B1 proteins (mutant and wild type) are aligned with those of the precursor polypeptides encoded by the human *CYP11B1* and *CYP11B2* genes. Six residues in red in the CYP11B1 mutant are the replacements of hydrophobic side chains with hydrophilic ones. Residues identical to those of the mutant are indicated by dots. Numbering starts with the amino-terminal methionine residue of the precursor polypeptides, which have the 24-residue mitochondrial targeting signal sequences (underlined).

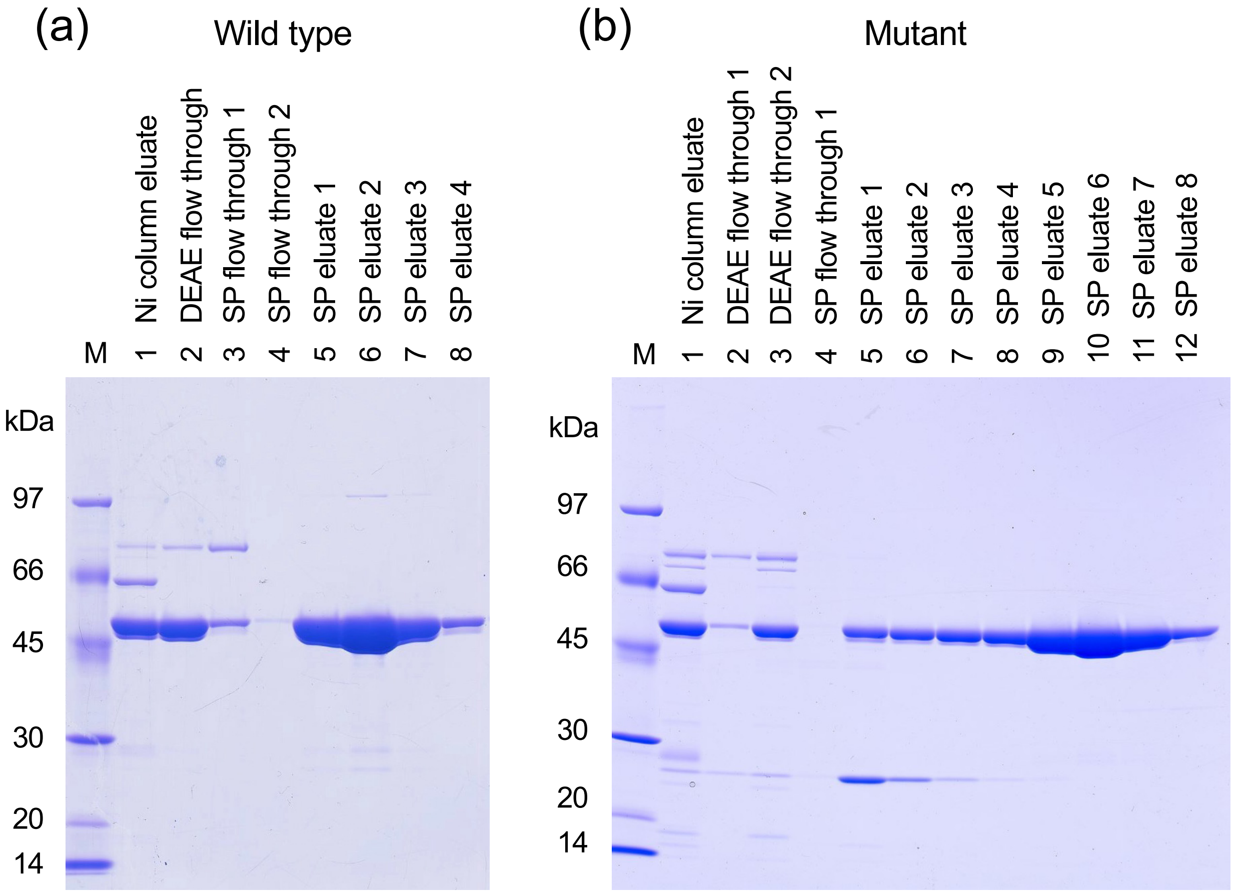

Figure S3. Purification of recombinant CYP11B1 proteins. (a) Wild type and (b) mutant CYP11B1 proteins were expressed using *E. coli* BL21(DE3) cells carrying pGro12 and purified as described under Materials and Methods. Column eluates were subjected to SDS-polyacrylamide gel electrophoresis, and proteins were stained with Coomassie Brilliant Blue. (a) Aliquots (2 μL) except for lane 3 (10 μL) and lane 6 (0.5 μL), and (b) aliquots (3 μL) were analyzed. M, molecular weight markers.

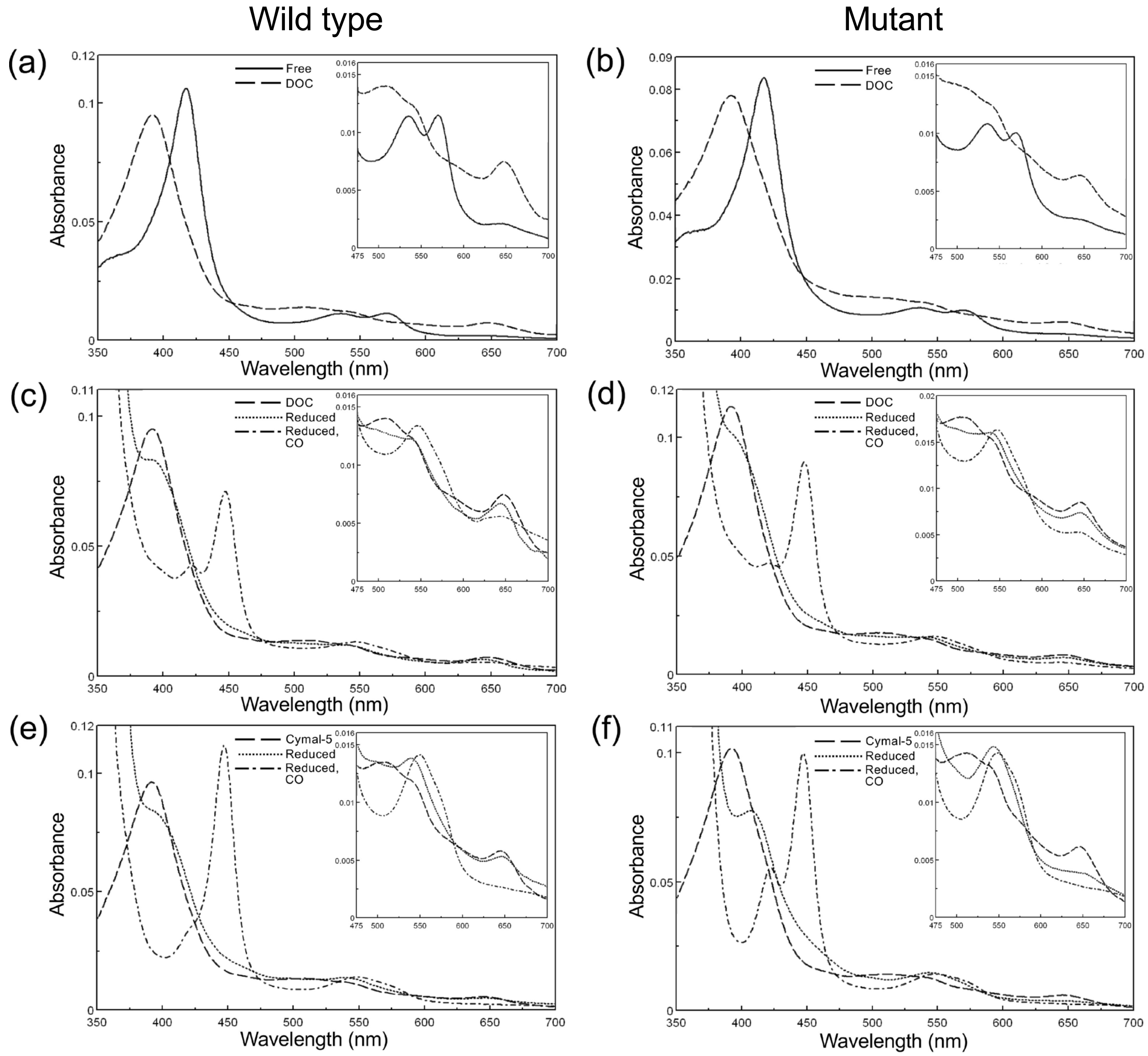

Figure S4. UV-visible spectra of purified CYP11B1 proteins. Absorption spectra of the wild type (a, c, and e) and the mutant (b, d, and f) of CYP11B1 were measured with procedures described under Materials and Methods. (a and b) Substrate-free (solid line) and DOC-bound ferric forms (dashed line). (c and d) DOC-bound ferric proteins (dashed line) were reduced by addition of sodium dithionite (dotted line), and then bubbled with pure CO gas (dashed-dotted line). (e and f) 5 mM Cymal-5 was added to substrate-free proteins (dashed line). The samples were reduced by addition of sodium dithionite (dotted line) and bubbled with CO gas (dashed-dotted line). The spectra in the range from 475 to 700 nm are also presented in insets.

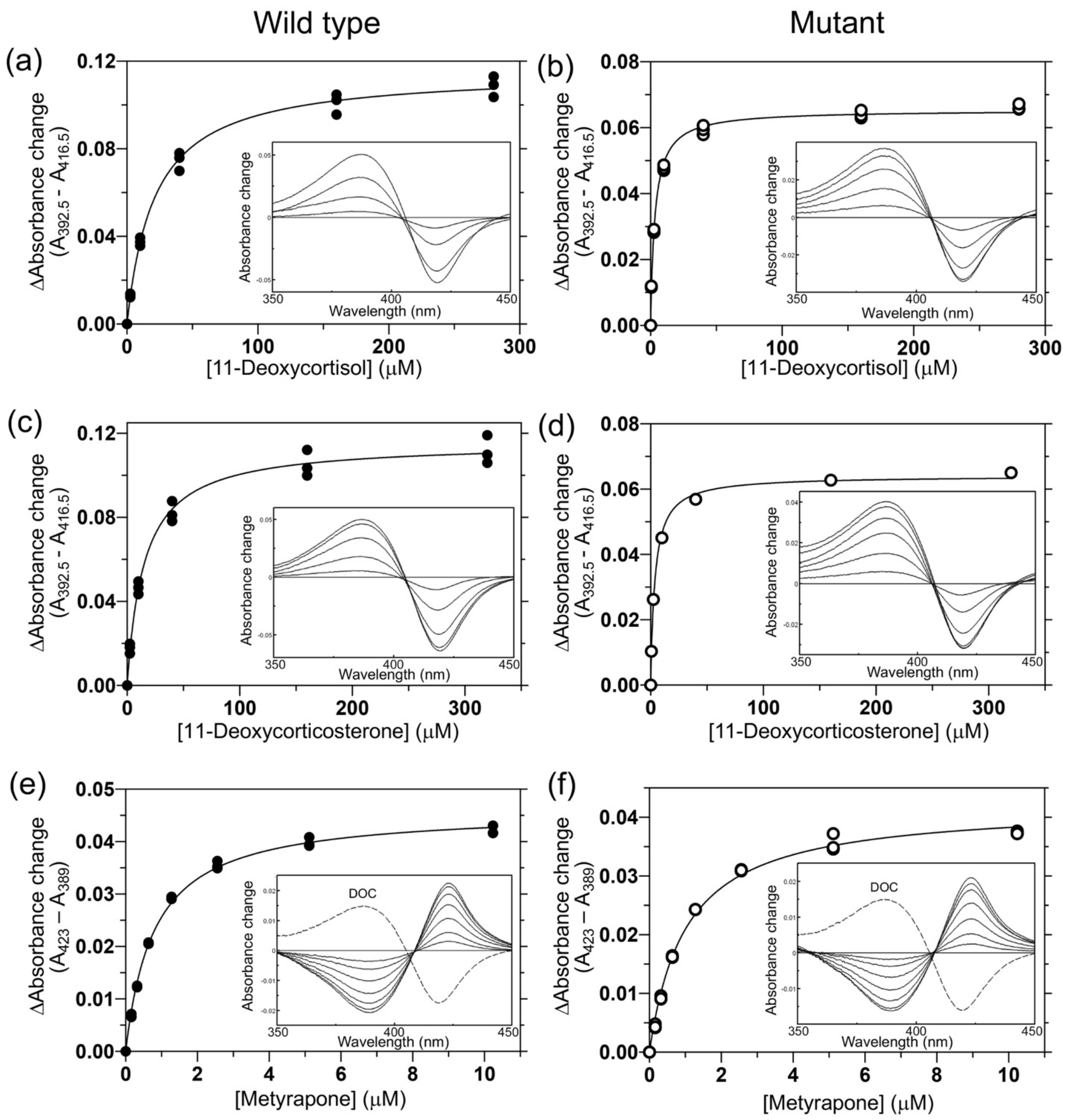

Figure S5. Binding of steroid and ligand. The wild type (a, c, and e) and the mutant (b, d, and f) of CYP11B1 were spectroscopically titrated with DOF (a and b) or DOC (c and d) and metyrapone in the presence of a saturating concentration of 320 μM DOC as competitor (e and f). Concentrations of the wild type were 1.4 μM in (a) and (c) and 0.35 μM in (e), and those of the mutant were 0.75 μM in (b) and (d) and 0.38 μM in (f). ΔAbsorbance change is a sum of absorbance changes at two wavelengths corresponding to the maximum and minimum in a difference spectrum. Raw values from separate experiments in triplicate are shown. Difference spectra measured with increasing concentrations of steroid or metyrapone are shown in insets. Dashed lines in insets of (e) and (f) show type-I spectral shift induced by addition of 320 μM DOC prior to titration.

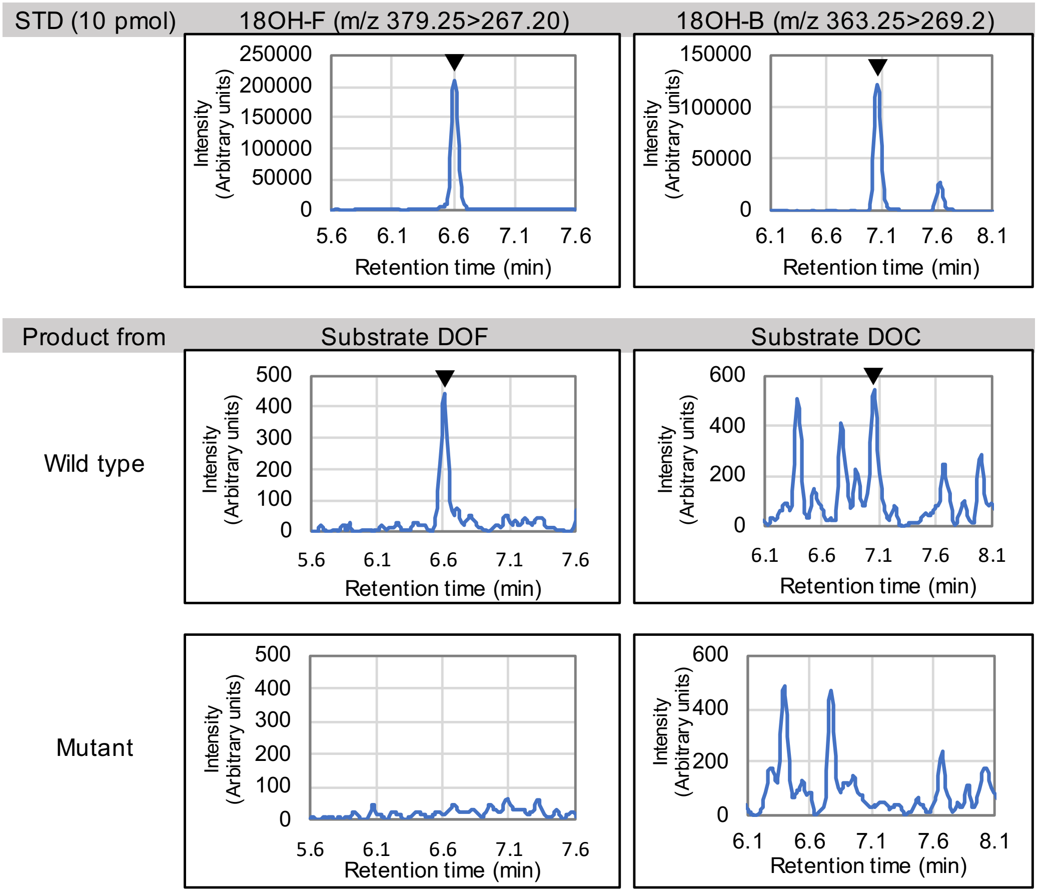

Figure S6. Analysis of 18-hydroxy cortisol and 18-hydroxy corticosterone. MS/MS analysis was performed as described under Materials and Methods. Standard 18-hydroxy cortisol (18OH-F) and 18-hydroxy corticosterone (18OH-B) gave signals shown in the upper panels. Profiles shown in the middle and lower panels were obtained with reaction products converted from 80 µM substrates 11-deoxycortisol (DOF) or 11-deoxycorticosterone (DOC) by the CYP11B1 wild type and mutant, respectively. The mutant has no catalytic activity of 11β,18-dihydroxylation.

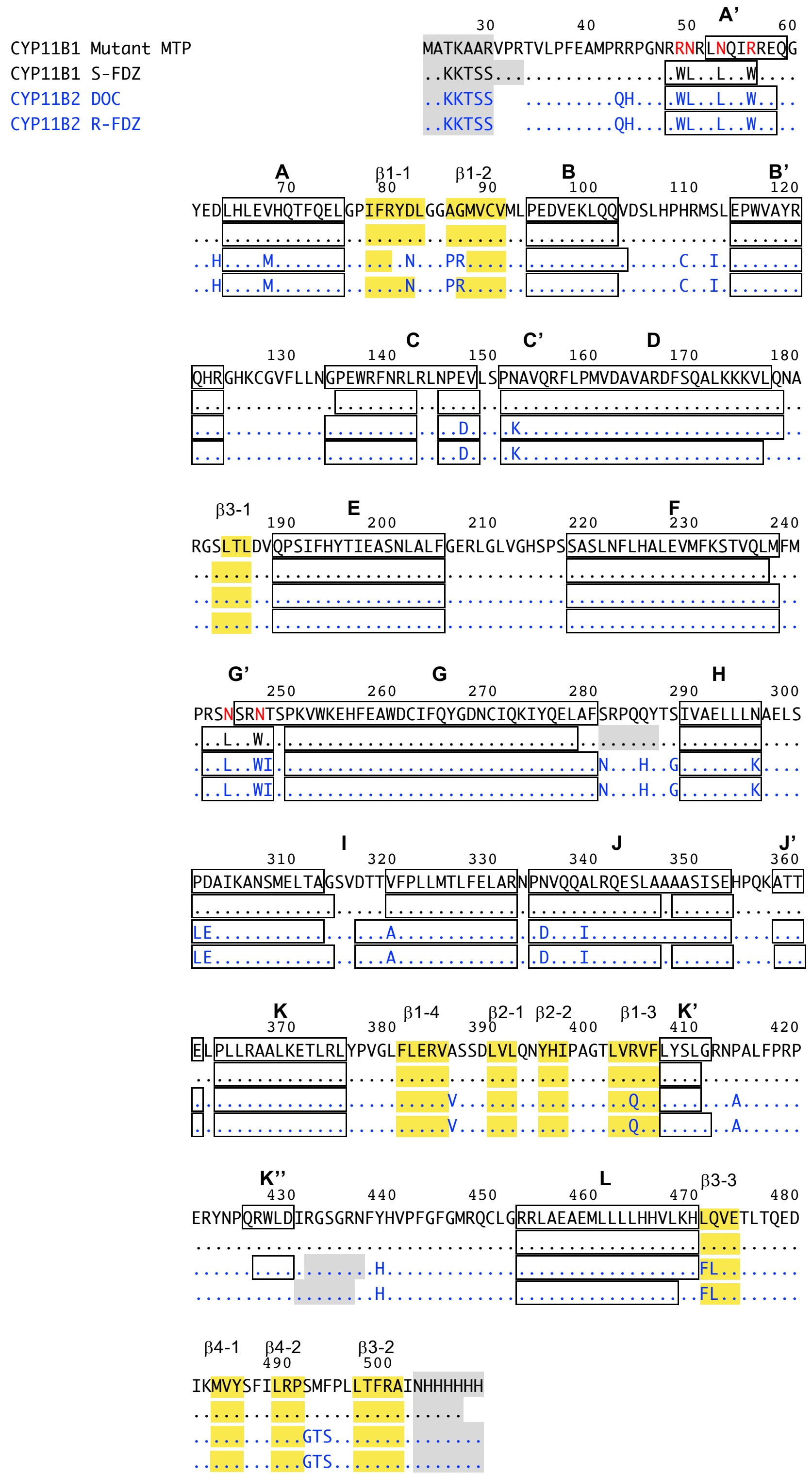

Figure S7. Comparison of secondary structures. Secondary structures of the recombinant human CYP11B1 mutant bound to metyrapone (MTP) are shown with those of the CYP11B1 wild type bound to (*S*)-fadrozole (S-FDZ) (PDB ID: 6M7X), CYP11B2 bound to DOC (PDB ID: 4DVQ), and CYP11B2 bound to (*R*)-fadrozole (R-FDZ) (PDB ID: 4FDH). Helices are boxed, and β-strands are highlighted. Residues with poor densities are shaded.

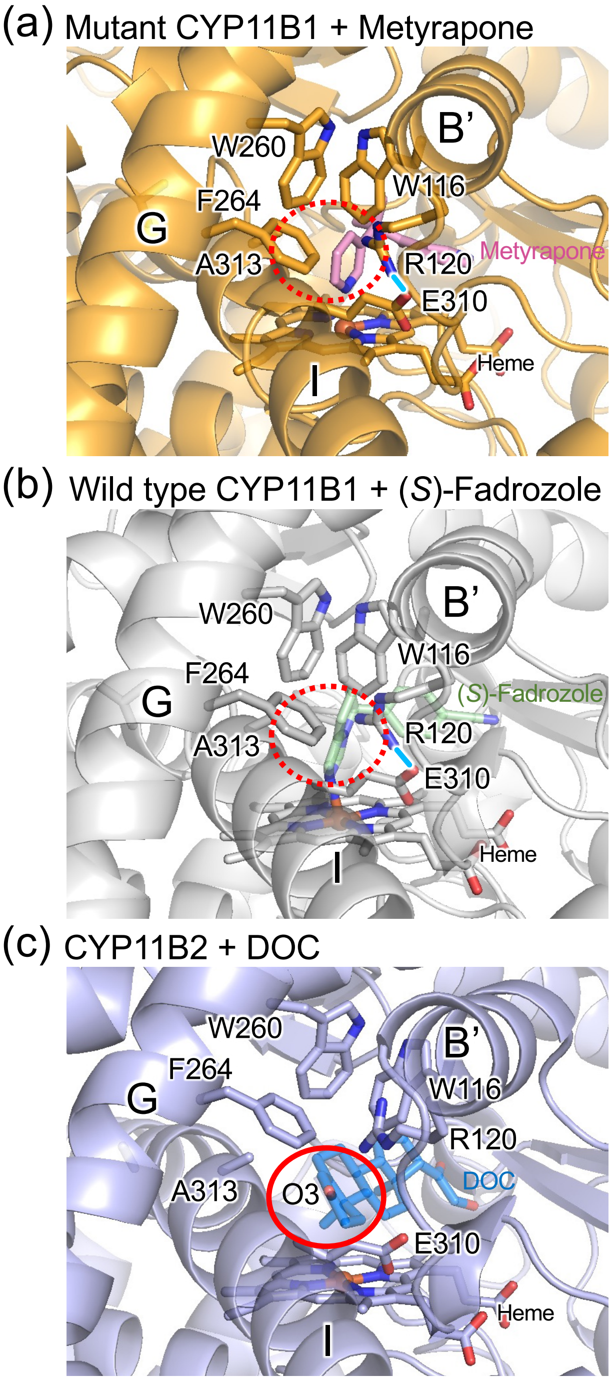

Figure S8. Arrangements of amino acid residues forming a putative water channel in CYP11B2 and those not forming in both CYP11B1s. Arrangements of the side chains of residues W116 and R120 (helix B'), W260 and F264 (helix G), and A313 and E310 (helix I) prevent formation of a water channel more strongly in (a) the CYP11B1 mutant compared to (b) the wild type. In (c) CYP11B2, the same set of the side chains forms a space for a water channel (red circle) that connects to the innermost area of the substrate-binding site. The position in both CYP11B1s corresponding to the channel formation in CYP11B2 is indicated by red dotted circle, and an ionic bond between R120 and E310 in both CYP11B1s is indicated by blue line.

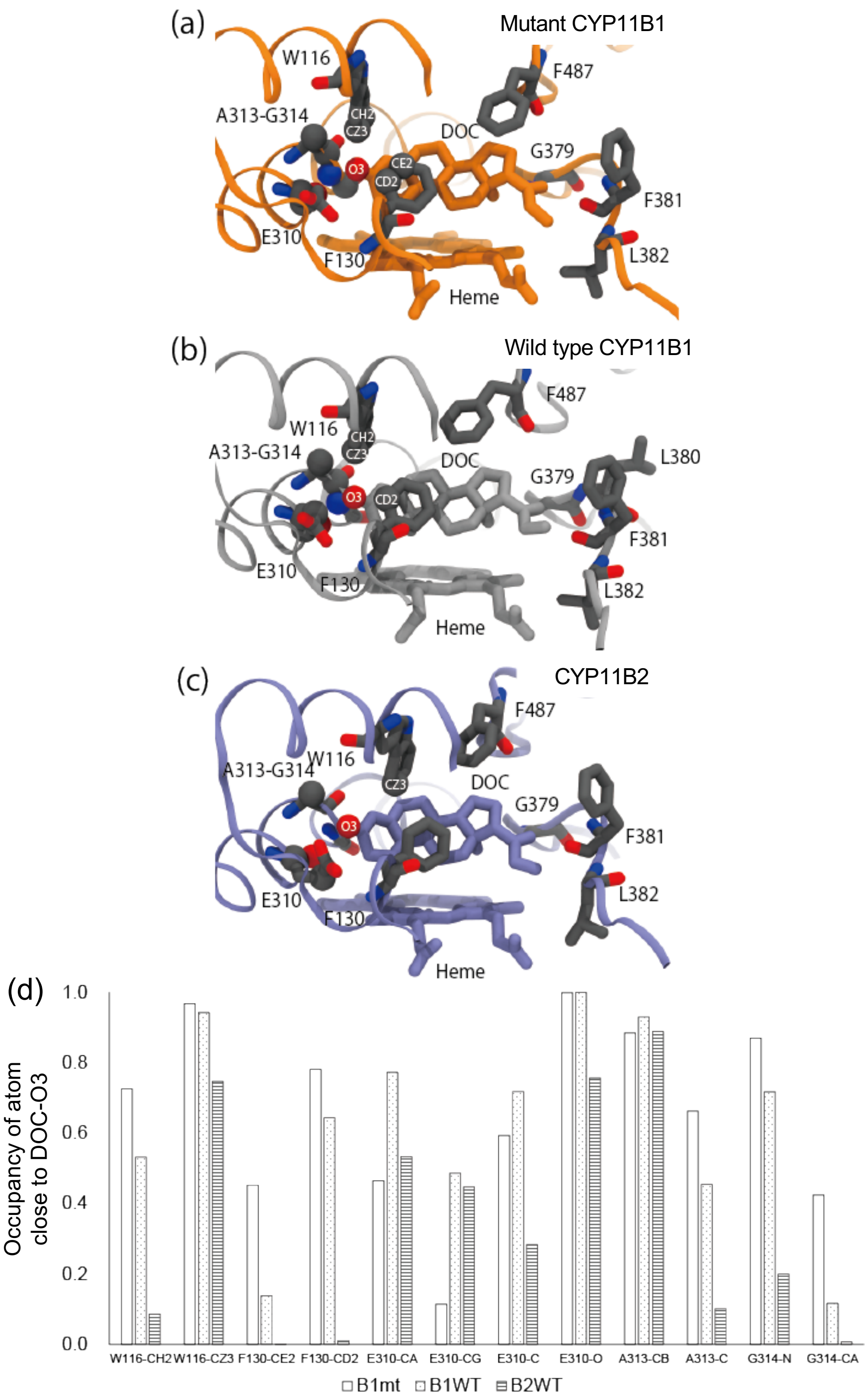

Figure S9. Amino acid residues close to DOC in MD simulation. MD snapshots show substrate-binding sites of DOC-bound forms of (a) mutant CYP11B1, (b) wild type CYP11B1, and (c) CYP11B2. Amino acid residues whose constituent atoms are located close (distance ≤4.0 Å and occupancy ≥0.5) to DOC (except for hydrogen atoms) are indicated by gray sticks. Residue atoms located close (distance ≤4.0 Å and occupancy ≥0.4) to O3 atom of DOC are indicated as balls. (d) Occupancy of residue atoms located close (distance ≤4.0 Å) to O3 atom of DOC.

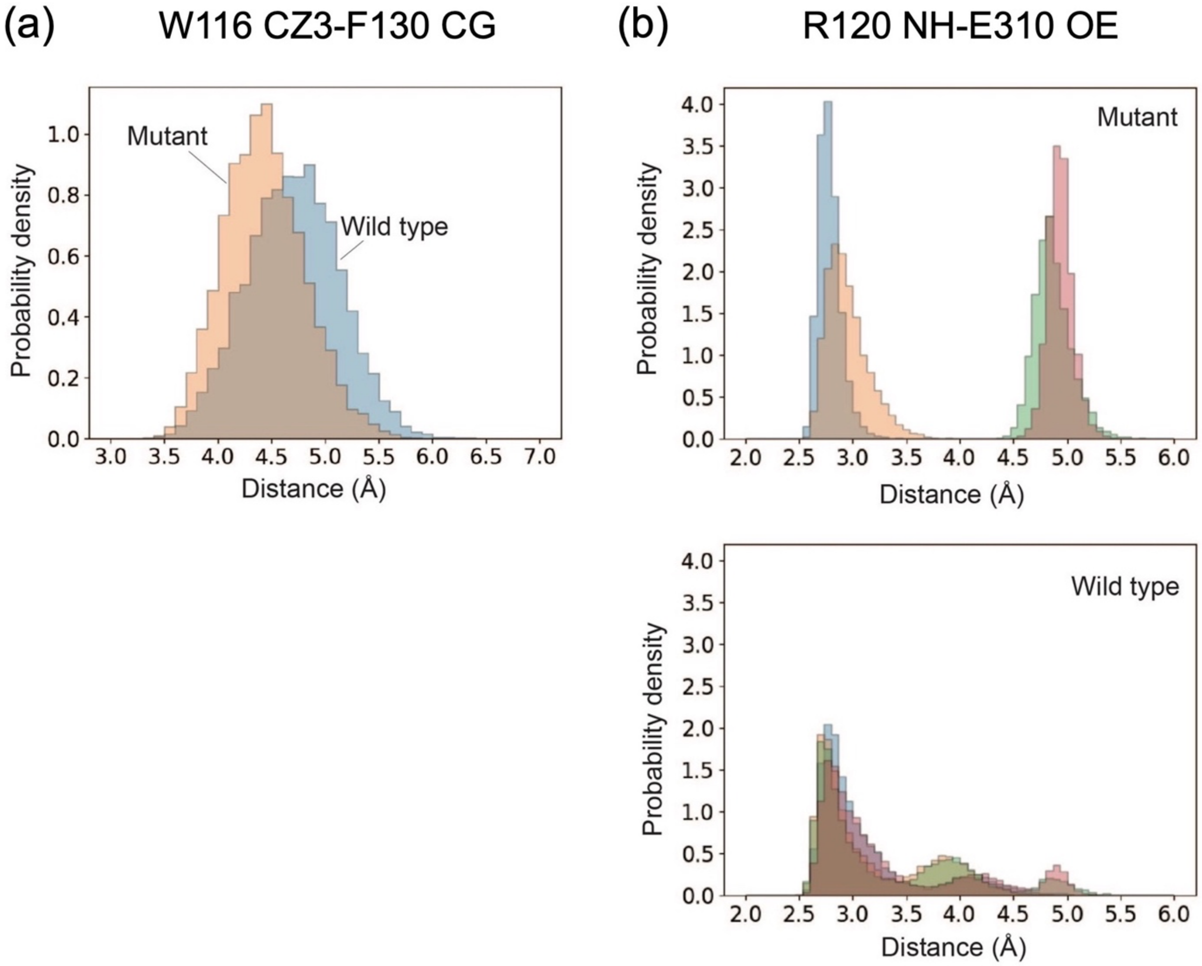

Figure S10. Distance distributions of W116-F130 and R120-E310 in metyrapone complexes of the CYP11B1 mutant and the wild type in MD simulation. (a) Distributions of the distance between the Cζ3 atom (CZ3) of W116 and the Cγ atom (CG) of F130 in the mutant (orange) and the wild type (blue). (b) Distributions of the four N-O distances between the side chains of R120 and E310 in the mutant (upper panel) and the wild type (lower panel). The two Nη atoms (NH1 and NH2) of R120 and the two Oε atoms (OE1 and OE2) of E310 were each distinguished by labeling them throughout the trajectories. The distributions are depicted in blue, orange, green, and pink in order of increasing average value. The four distributions in the mutant each have a single peak, whereas those in the wild type have two or three peaks.

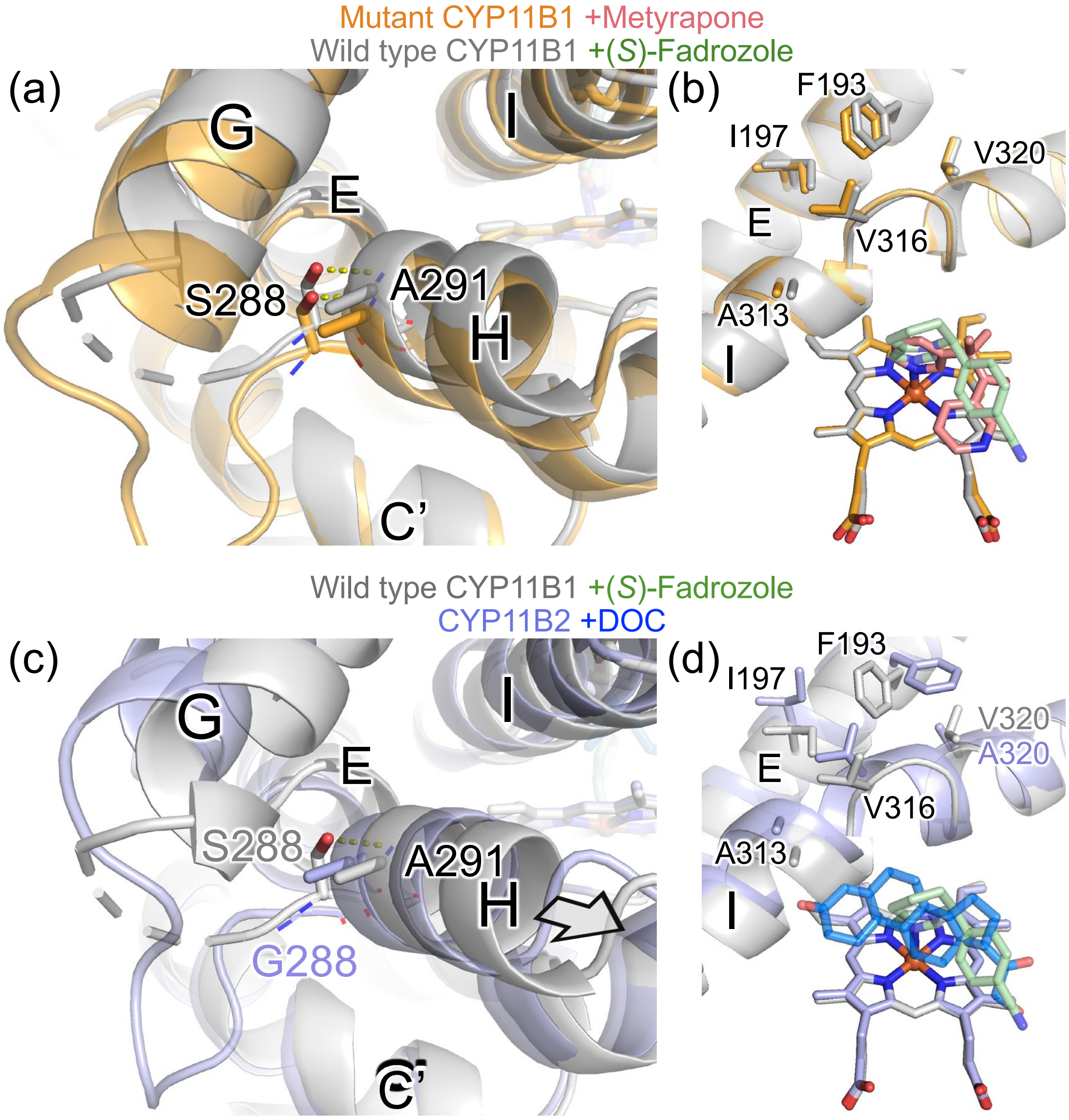

Figure S11. Possible involvement of isoform-specific residues at 288 and 320 in structural divergence between CYP11B1 and CYP11B2. (a and b) Superposition of metyrapone-bound CYP11B1 mutant and (*S*)-fadrozole-bound CYP11B1 wild type. (c and d) Superposition of (*S*)-fadrozole-bound CYP11B1 wild type and DOC-bound CYP11B2. The crystal structures are aligned using the same procedures as those for Figure 3. (a) The side chain of S288 of both CYP11B1s is located between helices E and H, which are almost tandemly arranged, and interacts with the amide nitrogen of A291 on helix H through hydrogen bonding (yellow dotted lines; distances 3.1 Å in the mutant and 3.3 Å in the wild type between acceptor and donor). (b) V320 on helix I of both CYP11B1s is located behind the substrate-binding site. The side chains of F193, I197, A313, V316, and V320 are in similar configurations between both CYP11B1s. (c) Interaction of S288 with A291 in wild type CYP11B1 contributes to the shifting of helix H outward (arrow), while G288 of CYP11B2 does not have such a structural effect on helix H. (d) Bulkier side chain of V320 in CYP11B1, compared to the side chain of A320 in CYP11B2, affects orientation of the side chains of F193 and I197 and causes upward distortion of the middle part including V316 of helix I. This changes the configuration of helix I with shifting the side chain of A313, leading to reduction of the space surrounding the C3 side of steroid in the substrate-binding site of CYP11B1.
